# Efficient Trifluoromethylation of Halogenated Hydrocarbons Using Novel [(bpy)Cu(O_2_CCF_2_SO_2_F)_2_] Reagent

**DOI:** 10.3390/molecules29122849

**Published:** 2024-06-14

**Authors:** Xiong Wu, Xin Qiu, Wenrun Lou, Shengxue Zhang, Chaoyi Zhang, Xiaoyu Ma, Chao Liu

**Affiliations:** 1 School of Chemical and Environmental Engineering, Shanghai Institute of Technology, 100 Haiquan Road, Shanghai 201418, China; 2 Shanghai-Sanming Engineering Research Center of Green Fluoropharmaceutical Technology, 25 Jingdong Road, Sanming 365004, China; 3 Key Laboratory of Organofluorine Chemistry, Shanghai Institute of Organic Chemistry, University of Chinese Academy of Sciences, 345 Lingling Road, Shanghai 200032, China

**Keywords:** trifluoromethylating agents, Chen’s reagent, [(bpy)Cu(O_2_CCF_2_SO_2_F)_2_]

## Abstract

This study introduces a novel trifluoromethylating reagent, [(bpy)Cu(O_2_CCF_2_SO_2_F)_2_], notable for not only its practical synthesis from cost-effective starting materials and scalability but also its nonhygroscopic nature. The reagent demonstrates high efficiency in facilitating trifluoromethylation reactions with various halogenated hydrocarbons, yielding products in good yields and exhibiting broad functional group compatibility. The development of [(bpy)Cu(O_2_CCF_2_SO_2_F)_2_] represents an advancement in the field of organic synthesis, potentially serving as a valuable addition to the arsenal of existing trifluoromethylating agents.

## 1. Introduction

Trifluoromethylating agents have garnered significant attention in the field of organic chemistry due to their pivotal role in introducing the trifluoromethyl (CF_3_) group into molecules [1,2,3,4,5,6,7,8]. This functional group is of paramount importance because of its ability to profoundly influence the physicochemical properties of compounds, making them crucial for the development of pharmaceuticals, agrochemicals, and materials science [9,10,11]. The CF_3_ group’s strong electron-withdrawing nature and high lipophilicity can dramatically alter a molecule’s metabolic stability, reactivity, and bioavailability. Consequently, the development of efficient and selective trifluoromethylating agents has been a focal point of research, aiming to achieve precise control over the incorporation of this moiety into target molecules. Recent advancements have seen the emergence of novel reagents and methodologies that facilitate the trifluoromethylation of a wide range of substrates under milder and more environmentally sustainable conditions [12,13,14,15,16,17,18,19,20,21,22,23,24,25,26,27,28,29]. For instance, the development of photoredox catalysis [12,13,14,15] and the use of less toxic and more readily available sources of the trifluoromethyl group [17,24] have marked significant milestones in this arena. As such, the ongoing research and development in this field continue to expand the toolkit of synthetic chemists, enabling the more widespread application of the trifluoromethyl group in organic synthesis.

**Scheme 1:**
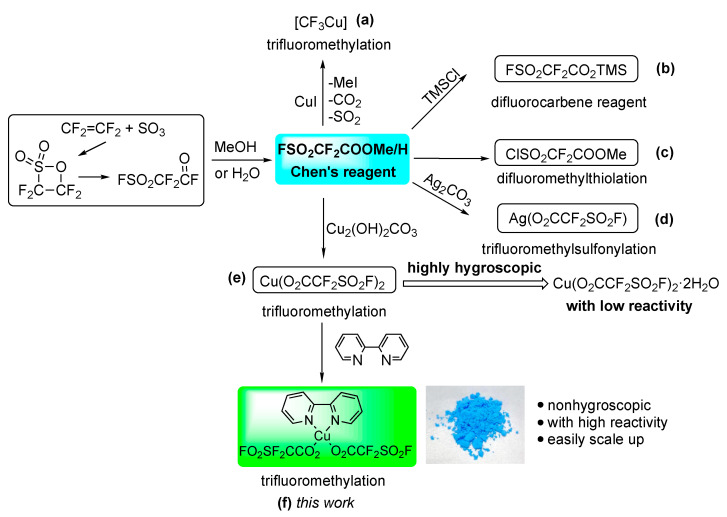
Representative fluoroalkylation reagents derived from Chen’s reagent (Shanghai Qinba Chemical Co., Ltd., Shanghai, China).

Among various strategies to incorporate the trifluoromethyl group into organic compounds, the introduction of Chen’s reagent, namely, fluorosulfonyldifluoroacetic methyl ester (FSO_2_CF_2_COOMe), has been a significant milestone, providing an efficient and cost-effective pathway for the trifluoromethylation of organic halides (Scheme 1a) [30,31,32,33]. This innovation marked the advent of catalytic trifluoromethylation, employing a novel strategy that utilizes the synergy between fluorine ion and in situ-generated difluorocarbene. However, this method exhibited limitations in terms of reaction diversity and the exploration of functional groups. Building on this pioneering work, our study has been directed towards the development and synthesis of a new generation of Chen’s reagent (Scheme 1b,c) [34,35], specifically targeting fluorosulfonyldifluoroacetate metal salts (Scheme 1d,e). [36,37,38] This endeavor aims to surpass the original reagent’s limitations by improving reaction conditions and expanding the reaction scope. By altering the metal ions in these salts, we have managed to finely tune their reactivity and reaction profiles, facilitating the incorporation of various fluorinated functional groups into a broad spectrum of organic molecules under more benign conditions. For instance, Cu(O_2_CCF_2_SO_2_F)_2_ has been recently identified as a more potent trifluoromethylation agent than FSO_2_CF_2_COOMe, capable of trifluoromethylating a wide range of hetero(aryl) iodides and benzyl bromides, even at ambient temperature [36]. Additionally, it has proven to be an effective and fast deoxofluorination agent for converting carboxylic acids into acyl fluorides [37]. Another versatile reagent, Ag(O_2_CCF_2_SO_2_F), facilitates the direct olefinic intermolecular radical trifluoromethylfluorosulfonylation reaction, with the in situ-generated SO_2_ from Ag(O_2_CCF_2_SO_2_F) decomposition being efficiently utilized [38].

The advancement brought about by Chen’s reagent and its derivatives has markedly propelled the domain of trifluoromethylation forward, providing innovative and efficient methodologies for such transformations. However, the practical application of these reagents, particularly the second-generation metal salts such as Cu(O_2_CCF_2_SO_2_F)_2_, has been hampered by their hygroscopic nature, which not only complicates handling and storage but also leads to low reactivity, potentially limiting their broader adoption in synthetic chemistry. In response to this limitation, our research is dedicated to devising novel approaches aimed at reducing the hygroscopic nature of these pivotal compounds, while preserving their chemical activity. We propose that the strategic integration of a ligand, which would occupy the coordination sites around the copper ion, could effectively exclude moisture from the compound’s structure, thereby yielding a copper complex with reduced moisture affinity (Scheme 1f). This approach not only aims to improve the handling and stability of Cu(O_2_CCF_2_SO_2_F)_2_ but also to explore the potential modulation of its reactivity by the presence of the ligand. Our selected ligand, 2,2′-bipyridine (bpy), is known for its capacity to form enduring complexes with copper ions, potentially leading to the creation of [(bpy) Cu(O_2_CCF_2_SO_2_F)_2_]. This novel complex is envisioned to act as a more durable and adaptable agent for trifluoromethylation, thereby improving its usability and broadening the scope of Cu(O_2_CCF_2_SO_2_F)_2_ applications in organic synthesis. Additionally, ligand introduction is anticipated to provide a new degree of control over the trifluoromethylation reaction’s reactivity and specificity, facilitating more refined and targeted modifications of organic structures. Within this context, we detail our findings.

## 2. Results

### 2.1. Synthesis of Fluorosulfonyl Difluoroacetic Acid Copper Complex [(bpy)Cu(O_2_CCF_2_SO_2_F)_2_]

**Scheme 2:**
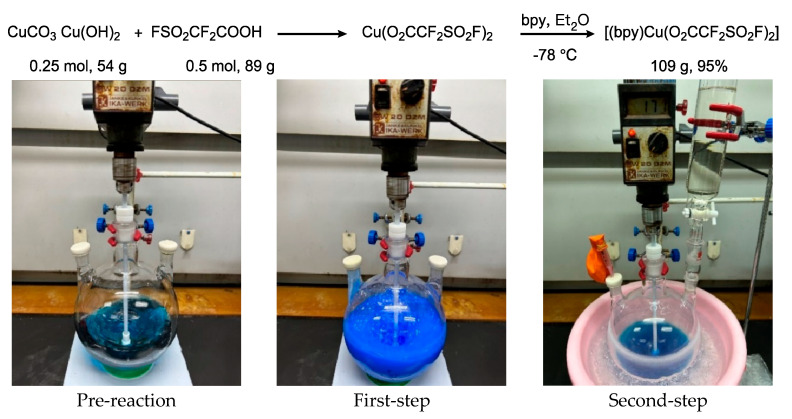
One-pot scalable synthesis of [(bpy)Cu(O_2_CCF_2_SO_2_F)_2_]. Reaction conditions: CuCO_3_Cu(OH)_2_ (0.25 mol), FSO_2_CF_2_COOH (0.5 mol), bpy (0.2 mol) in redistilled Et_2_O (300 mL).

We initially attempted to synthesize fluorosulfonyl difluoroacetic acid copper complex [(bpy)Cu(O_2_CCF_2_SO_2_F)_2_] by one-pot reaction from fluorosulfonyl difluoroacetic acid, copper species, and 2,2′-bipyridine (bpy) at room temperature (Table 1, entries 1 and 2). Unfortunately, no target compound was generated, and the system was observed to release gas upon addition of the ligand. This may be attributed to the potential reaction of fluorosulfonyl difluoroacetic acid with 2,2′-bipyridine, which leads to the decomposition of fluorosulfonyl difluoroacetic acid and fails to produce the corresponding intermediate Cu(O_2_CCF_2_SO_2_F)_2_ (**1**) and target compound [(bpy)Cu(O_2_CCF_2_SO_2_F)_2_] (**2**). Subsequently, we chose dry **1** as substrate to react with 2,2′-bipyridine at room temperature, resulting to no formation of the desired product **2** (entry 3). Based on our previous work [36], we found that low temperature can suppress the rapid decomposition of Cu(O_2_CCF_2_SO_2_F)_2_. Therefore, we assessed the impact of temperature on the outcomes during the ligand addition process (entries 4–7). To our delight, the desired product **2** was obtained with a 95% yield when the temperature was lowered to −78 °C (entry 5). Notably, substituting the ligand with tert-butyl-substituted 2,2′-bipyridine under the optimal conditions afforded the corresponding complex [(^t^Bu-bpy)Cu(O_2_CCF_2_SO_2_F)_2_], and its structure was confirmed by X-ray crystallographic study (see ESI). Further screening of the ligand amount revealed that an increase in ligand equivalents corresponded to a decrease in yield. With 0.8 equivalents of the ligand, the yield stabilized at 96% (entries 8–11). In an effort to streamline the reaction process, we attempted to react the undried Cu(O_2_CCF_2_SO_2_F)_2_ with the ligand, which, surprisingly, did not affect the outcome of the reaction (entry 12). On the basis of optimization mentioned above, the optimized conditions for the preparation of [(bpy)Cu(O_2_CCF_2_SO_2_F)_2_] were set as follows: Cu(O_2_CCF_2_SO_2_F)_2_ (1.0 equiv), ligand (0.8 equiv), −78 °C to room temperature in redistilled Et_2_O under Ar atmosphere. To further simplify the operation, we decided to carry out one-pot scalable synthesis of the desired product **2** under the optimal reaction conditions (Scheme 2). The reaction was scaled up on a 0.25 mol scale to give 109 g of target reagent [(bpy)Cu(O_2_CCF_2_SO_2_F)_2_]. The purification process is notably straightforward, necessitating only simple filtration, thereby streamlining the workflow for laboratory and potential industrial applications.

### 2.2. [(bpy)Cu(O_2_CCF_2_SO_2_F)_2_] as a Trifluoromethylating Reagent

In 2016, our group successfully achieved trifluoromethylation of aryl halides using Cu(O_2_CCF_2_SO_2_F)_2_ as a trifluoromethylating agent, delivering excellent yields at room temperature [36]. Encouraged by this result, we hypothesized that [(bpy)Cu(O_2_CCF_2_SO_2_F)_2_], as a derivative of Cu(O_2_CCF_2_SO_2_F)_2_, might also facilitate nucleophilic trifluoromethylation reactions with aryl halides. An initial attempt was carried out by using 1-iodonaphthalene (**3a**) as the model substrate and [(bpy)Cu(O_2_CCF_2_SO_2_F)_2_] as trifluoromethyl reagent, with Cu serving as a reductant in anhydrous DMF at room temperature for 3 h. Gratifyingly, the target compound was obtained with a ^19^FNMR yield of 23% (Table 2, entry 1). In an effort to improve the reaction yield, the impact of the amount of [(bpy)Cu(O_2_CCF_2_SO_2_F)_2_] and Cu on the reaction outcome was firstly investigated. The results showed that when increasing the amount of [(bpy)Cu(O_2_CCF_2_SO_2_F)_2_] and Cu to 2.0 equivalents, the yield of the target compound increased to 62% (entry 2). Inspired by Shen’s work [18], we tried to change the reaction temperature to improve the yield of **4a**. The results showed that when the reaction temperature was increased to 60 °C, the desired product **4a** was obtained in 85% yield and continued to increase the temperature; the yield decreased slightly (entries 3–5). Further screening of other solvents showed that NMP was the best choice for this transformation (entries 6). In contrast, the target product was not observed when MeCN was used as a solvent, which may have been due to the fact that the complexes were not completely decomposed in acetonitrile (entry 7). Detailed screening information on the reaction conditions can be found in the ESI.

**Scheme 3:**
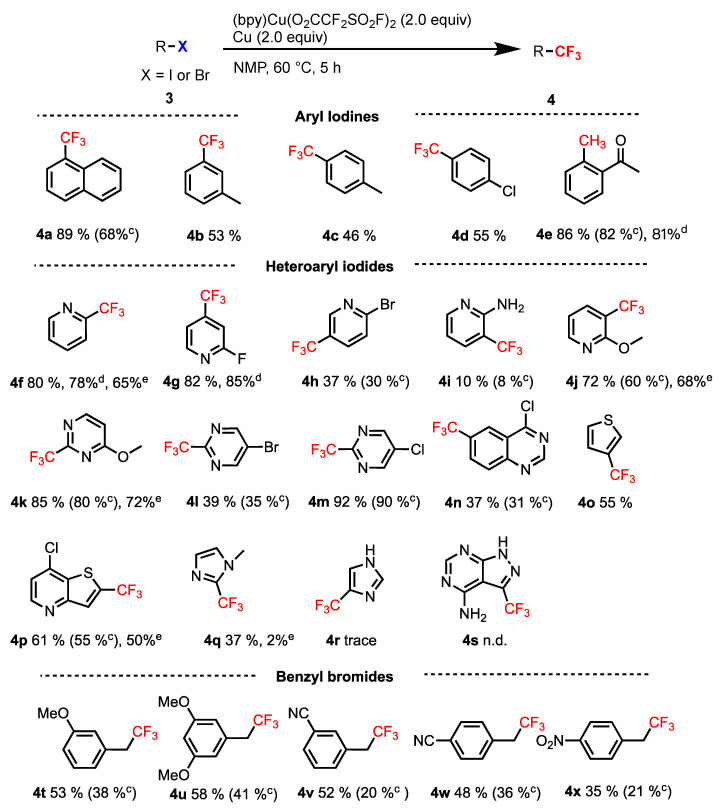
Scope of the trifluoromethylation of various halogenated hydrocarbons ^a,b^. ^a^ General reaction conditions: aryl halogen (0.4 mmol, 1.0 equiv.), NMP (6 mL), Ar atmosphere, 60 °C, 5 h. ^b^ Yields were determined by ^19^F NMR with trifluorotoluene as internal standard. ^c^ Isolated yield. ^d^ [(bpy)Cu(O_2_CCF_2_SO_2_F)_2_] was exposed to the air for 3 days and used as trifluoromethylating reagent. ^e^ [(^t^Bu-bpy)Cu(O_2_CCF_2_SO_2_F)_2_] was used as trifluoromethylating reagent.

With the optimal reaction conditions successfully established, we turned our focus to studying the scope of the reaction. As shown in Scheme 3, a variety of aryl iodides (**3a**–**e**) could be subjected to trifluoromethylation under mild conditions, giving the corresponding trifluoromethylated products in good to excellent yields. In general, electron-deficient aryl iodides (**3e**) demonstrated better reactivity and yields than electron-rich aryl iodides (**3b**–**d**). We also explored the scope of the trifluoromethylation reaction by utilizing heteroaryl iodides with functional group, such as iodopyridenes, iodopyrimidines, iodoquinazoline, 2-iodothieno[3,2-b]pyridine, 1-methyliodoimidazole, and iodothiophene, and these could work smoothly under our optimal conditions (**3f**–**q**). However, the preparation of 4-(trifluoromethyl)-1*H*-imidazole (**4r**) and 3-(trifluoromethyl)-1*H*-pyrazolo[3,4-*d*]pyrimidin-4-amine (**4s**) was not suitable for this method. In addition, a series of benzyl bromide compounds could also be converted to their corresponding trifluoromethyl compounds in moderate yields (**4t**–**x**). In order to prove its non-hygroscopicity, the performance of [(bpy)Cu(O_2_CCF_2_SO_2_F)_2_] as a trifluoromethylation reagent was further explored by exposing it to air for 3 days, and the results showed that it has good stability and reactivity (**4e**, **4f**, **4g**). Furthermore, we compared the efficiency of [(bpy)Cu(O_2_CCF_2_SO_2_F)_2_] and [(^t^Bu-bpy)Cu(O_2_CCF_2_SO_2_F)_2_] as trifluoromethylation reagents. The results showed that the reactivity of [(bpy)Cu(O_2_CCF_2_SO_2_F)_2_] was higher than [(^t^Bu-bpy)Cu(O_2_CCF_2_SO_2_F)_2_] (**4f**, **4j**, **4k**, **4p**, **4q**).

**Scheme 4:**
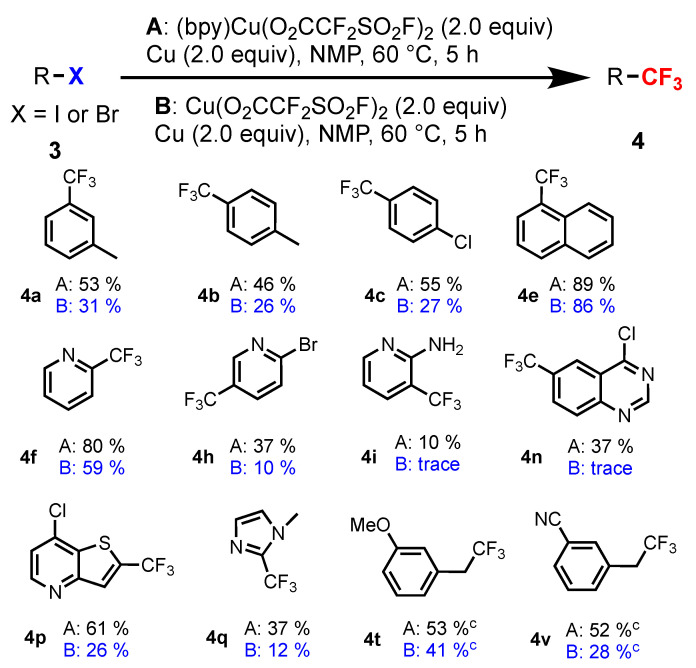
Comparison of trifluoromethylating reagent [(bpy)Cu(O_2_CCF_2_SO_2_F)_2_] with Cu(O_2_CCF_2_SO_2_F)_2_ on various halogenated hydrocarbons ^a,b^. ^a^ General reaction conditions: aryl halogen (0.4 mmol, 1.0 equiv.), NMP (6 mL), Ar atmosphere, 60 °C, 5 h. ^b^ Yields were determined by ^19^F NMR with trifluorotoluene as internal standard. ^c^ Using benzyl bromide as substrates.

Additionally, we conducted a detailed comparative analysis of the reactivity profiles of the newly synthesized trifluoromethylating reagent, [(bpy)Cu(O_2_CCF_2_SO_2_F)_2_], and the previously established reagent, Cu(O_2_CCF_2_SO_2_F)_2_, across a spectrum of halogenated hydrocarbons. The experimental outcomes, as delineated in Scheme 4, demonstrate that in most cases, [(bpy)Cu(O_2_CCF_2_SO_2_F)_2_] exhibits superior reactivity, coupled with enhanced yield, relative to its counterpart, Cu(O_2_CCF_2_SO_2_F)_2_. This comparative evaluation not only underscores the efficacious nature of the [(bpy)Cu(O_2_CCF_2_SO_2_F)_2_] reagent but also highlights its potential utility in facilitating more efficient trifluoromethylation reactions within the realm of organic synthesis.

## 3. Materials and Methods

### 3.1. General Information of Materials and Instruments

The NMR spectra were obtained on a 400 MHz spectrometer using CDCl_3_ as deuterated solvent, with proton, carbon, and fluorine resonances at 400 MHz, 100 MHz, and 376 MHz, respectively. Chemical shifts were reported in parts per million (ppm) relative to residual chloroform (7.26 ppm) or TMS as an internal standard (δ_TMS_ = 0 ppm) for ^1^H and ^13^C NMR spectra and benzotrifluoride as an external standard (negative for upfield) for ^19^F NMR spectra. The following abbreviations were used to explain the multiplicities: s = singlet, d = doublet, t = triplet, q = quartet, m = multiplet. The NMR yield was determined by ^19^F NMR using benzotrifluoride (^19^F NMR: δ-63.0 ppm) as an internal standard before working up the reaction. GC-MS (EI) data were determined on an Agilent 5975C. LRMS (EI) and HRMS (EI) data were tested on a Water Micromass GCT Premier. HRMS (ESI) data was tested on a Thermo Fischer Scientific LTQ FT Ultra instrument in DART-positive mode. X-ray powder diffraction (XRD) experiments were conducted on TD-3500. All reagents were used as received from commercial sources or prepared as described in the literature. Solvents were freshly dried and degassed according to the purification handbook Purification of Laboratory Chemicals before use. Flash column chromatography was carried out using 300–400 mesh silica gel.

### 3.2. General Procedure for the Synthesis of [(bpy)Cu(O_2_CF_2_SO_2_F)_2_]

An oven-dried 1 L three-necked round-bottom flask equipped with a stir bar was charged with redistilled Et_2_O (300 mL) and Cu_2_(OH)_2_CO_3_ (54 g, 0.25 mol). FSO_2_CF_2_COOH (89 g, 0.5 mol) was added dropwise during a period of 1 h, and the carbon dioxide produced during the reaction process was removed via a bubbler. The reaction mixture was stirred at room temperature for 24 h. The resulting reaction mixture was filtered via Celite pad. After cooling the ether solution of Cu(O_2_CCF_2_SO_2_F)_2_ to −40 °C, bpy ether solution (31 g, 0.2 mol) was slowly added to the system. The reaction mixture was stirred at room temperature for 30 min and filtered to obtain blue solid [(bpy)Cu(O_2_CCF_2_SO_2_F)_2_] (109 g, 96%).

### 3.3. General Procedure for the Synthesis of Compounds *(**4a**–**x**)*

Aryl iodide (0.4 mmol), Cu (51 mg, 0.8 mmol), and [(bpy)Cu(O_2_CCF_2_SO_2_F)_2_] (459 mg, 0.8 mmol) were added to an oven-dried Schlenk tube equipped with a magnetic rotor under Ar atmosphere, and then NMP (6 mL) was added to the system. The mixture was stirred at 60 °C for 5 h. Trifluorotoluene was added into the reaction mixture as an internal standard, and the yield of the desired product was measured by ^19^F NMR. The reaction mixture was then subjected to filtration. The filtrate was washed with water (5 mL) and brine (5 mL), followed by drying over anhydrous Na_2_SO_4_, and then filtered. The filtrate was evaporated under reduced pressure. The resulting crude material was purified by flash column chromatography on silica gel with an ethyl acetate/petroleum ether (EA/PE = 15:1, *v*/*v*) mixture as eluent to afford the desired trifluoromethylated product.

## 4. Conclusions

In summary, we introduced a novel trifluoromethylating agent, [(bpy)Cu(O_2_CCF_2_SO_2_F)_2_], characterized by its synthesis from readily available and economical starting materials on a large scale. This agent is distinguished by its nonhygroscopic nature, which enhances its ease of handling and storage. Moreover, this new trifluoromethylating reagent can efficiently conduct the trifluoromethylation reactions with various halogenated hydrocarbons in good to excellent yields with good functional group compatibility. We anticipate that the development of [(bpy)Cu(O_2_CCF_2_SO_2_F)_2_] will be a good complement to the well-established trifluoromethylating reagent and may have a widespread application in organic synthesis. Our ongoing investigations aim to expand on the synthesis and application of [(bpy)Cu(O_2_CCF_2_SO_2_F)_2_], highlighting its promising role in future organic chemical research.

## Figures and Tables

**Table 1:** Synthesis of anhydrous [(bpy)Cu(O_2_CCF_2_SO_2_F)_2_] ^a^.

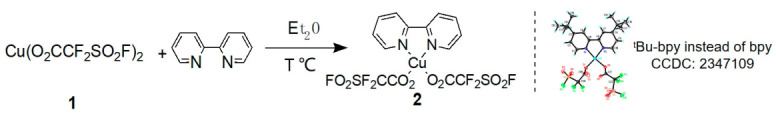				
Entry	[Cu]	Equivalent	T (°C)	Yield (%) ^b^
1	Cu(OH)_2_	1:1	r.t.	0 ^c^
2	Cu_2_(OH)_2_CO_3_	1:1	r.t.	0 ^c^
3	dry Cu(O_2_CCF_2_SO_2_F)_2_	1:1	r.t.	0
4	dry Cu(O_2_CCF_2_SO_2_F)_2_	1:1	−196	85
5	dry Cu(O_2_CCF_2_SO_2_F)_2_	1:1	−78	95
6	dry Cu(O_2_CCF_2_SO_2_F)_2_	1:1	−40	96
7	dry Cu(O_2_CCF_2_SO_2_F)_2_	1:1	0	0
8	dry Cu(O_2_CCF_2_SO_2_F)_2_	1:1.2	−78	85
9	dry Cu(O_2_CCF_2_SO_2_F)_2_	1:1	−78	97
10	dry Cu(O_2_CCF_2_SO_2_F)_2_	1:0.9	−78	96 ^d^
11	dry Cu(O_2_CCF_2_SO_2_F)_2_	1:0.8	−78	96 ^d^
12	undried Cu(O_2_CCF_2_SO_2_F)_2_	1:0.8	−78	96 ^d^

^a^ Reaction conditions: Cu(O_2_CCF_2_SO_2_F)_2_ (0.2 mmol, 1.0 equiv), bpy, redistilled Et_2_O (2 mL), Ar atmosphere, 30 min. ^b^ Isolated yields are reported. ^c^ The fluorosulfonyl difluoroacetic acid (1.0 equiv) was used as a starting material. ^d^ The yield calculation is based on bpy.

**Table 2:** Conditional filter ^a^.

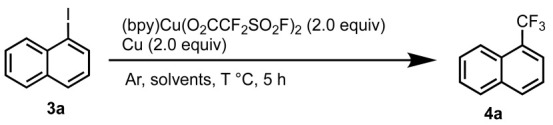					
Entry	[Cu] (Equiv)	Cu (Equiv)	T (°C)	Solvent	Yield (%) ^b^
1	1.5	1.5	−196~RT	dry DMF	23
2	2.0	2.0	−196~RT	dry DMF	62
3	2.0	2.0	RT	dry DMF	53
4	2.0	2.0	RT~60	dry DMF	85
5	2.0	2.0	RT~80	dry DMF	82
6	2.0	2.0	RT~60	NMP	89
7	2.0	2.0	RT~60	MeCN	N.D.

^a^ Reaction conditions: 1-iodonaphthalene (0.2 mmol, 1.0 equiv), (bpy)Cu(O_2_CCF_2_SO_2_F)_2_, Cu, in solvent (2 mL) under Ar atmosphere. ^b^ Yields were determined by ^19^F NMR with trifluorotoluene as internal standard.

## Data Availability

The data underlying this study are available in the published article and its Supplementary Materials.

## Supplementary Materials

The following supporting information can be downloaded at: https://www.mdpi.com/article/10.3390/molecules29122849/s1, Figure S1. XRD spectrum of [(bpy)Cu(O_2_CCF_2_SO_2_F)_2_]; Figure S2. XRD spectrum of [(^t^Bu-bpy)Cu(O_2_CCF_2_SO_2_F)_2_]; Figure S3. ESI-MS spectrum of [(bpy)Cu(O_2_CCF_2_SO_2_F)_2_]; Table S1. Crystal data and structure refinement for [(^t^Bu-bpy)Cu(O_2_CCF_2_SO_2_F)_2_]; Table S2. Screening on the temperature during the ligand addition process; Table S3. Screening on the equivalent of Cu(O_2_CCF_2_SO_2_F)_2_ and ligand; Table S4. Screening on the reaction time; Table S5. Screening on the reaction temperature and equivalent of [(bpy)Cu(O_2_CCF_2_SO_2_F)_2_] and Cu; Table S6. Screening on solvent. References [36,39,40,41,42,43,44,45] are cited in the Supplementary Materials.
