# Supplementary material for: Efficient Trifluoromethylation of Halogenated Hydrocarbons Using Novel [(bpy)Cu(O2CCF2SO2F)2] Reagent
Source: Molecules. 2024 Jun 14;29(12):2849. doi: 10.3390/molecules29122849 (PMC11206303; doi:10.3390/molecules29122849)
Supplement: Supplementary file 1 [file molecules-29-02849-s001.zip › molecules-2986093-supplementary.pdf]

Supporting Information

Efficient Trifluoromethylation of Halogenated Hydrocarbons Using Novel [(bpy)Cu($O_2CCF_2SO_2F$)$_2$] Reagent

Xiong Wu [1,†], Xin Qiu [1,†], Wenrun Lou [1], Shengxue Zhang [1], Chaoyi Zhang [1], Xiaoyu Ma [1,2,*] and Chao Liu [1,2,3,*]

1 School of Chemical and Environmental Engineering, Shanghai Institute of Technology, 100 Haiquan Road, Shanghai 201418, China

2 Shanghai-Sanming Engineering Research Center of Green Fluoropharmaceutical Technology, 25 Jingdong Road, Sanming 365004, China

3 Key Laboratory of Organofluorine Chemistry, Shanghai Institute of Organic Chemistry, University of Chinese Academy of Sciences, 345 Lingling Road, Shanghai 200032, China

* Correspondence: maxiaoyu@sit.edu.cn (X.M.); chaoliu@sit.edu.cn (C.L.)

† These authors contributed equally to this work.

Table of contents

I. Procedures for the preparation of [(^{t}Bu-bpy)Cu($O_2CF_2SO_2F$)$_2$] S2

II. Characterization of [(bpy)Cu($O_2CCF_2SO_2F$)$_2$] and [(^{t}Bu-bpy)Cu($O_2CF_2SO_2F$)$_2$] S3

III. Screening reaction condictions..................S8

IV. Analytical data for compounds 4a–4x S11

V. References S20

VI. Copies of ^{1}H NMR, ^{19}F NMR and ^{13}C NMR spectra..................S22

I. Procedures for the preparation of [(^{t}Bu-bpy)$Cu(O_2CF_2SO_2F)_2$]

$$Cu(OH)_2CO_3 + FSO_2CF_2COOH \xrightarrow[24\ h]{Et_2O,\ RT} Cu(O_2CCF_2SO_2F)_2 \xrightarrow[Et_2O,\ -78°C\ -\ RT]{^tBu\text{-}bpy} (^tBu\text{-}bpy)Cu(O_2CCF_2SO_2F)_2$$

An oven-dried 500 mL three-necked round-bottom flask equipped with a stir bar was charged with redistilled Et_2O (200 mL) and $Cu_2(OH)_2CO_3$ (32 g, 0.2 mol). FSO_2CF_2COOH (71 g, 0.4 mol) was added dropwise during a period of 1 h, and the carbon dioxide produced during the reaction process was removed by a bubbler. The reaction mixture was stirred at room temperature for 24 h. The resulting reaction mixture was filtered via Celite pad. After cooling the ether solution of $Cu(O_2CCF_2SO_2F)_2$ to -40 °C, 4,4 '- ditert-butyl-2,2' - bipyridine ether (43 g, 0.16 mol) was added slowly. The reaction mixture was stirred at room temperature for 30 min, and filtered to get blue solid [(^{t}Bu-bpy)$Cu(O_2CF_2SO_2F)_2$] (102 g, 93%).

II. Characterization of [(bpy)Cu($O_2CCF_2SO_2F$)$_2$] and [(^{t}Bu-bpy)Cu($O_2CF_2SO_2F$)$_2$]

(a) XRD of [(bpy)Cu($O_2CCF_2SO_2F$)$_2$] and [(^{t}Bu-bpy)Cu($O_2CF_2SO_2F$)$_2$]

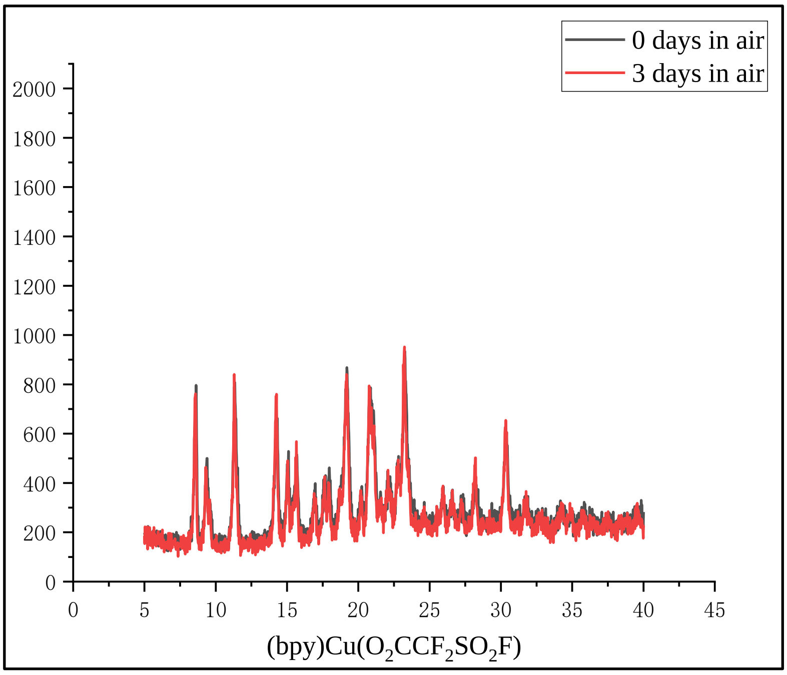

Figure S1 XRD spectrum of [(bpy)Cu($O_2CCF_2SO_2F$)$_2$]

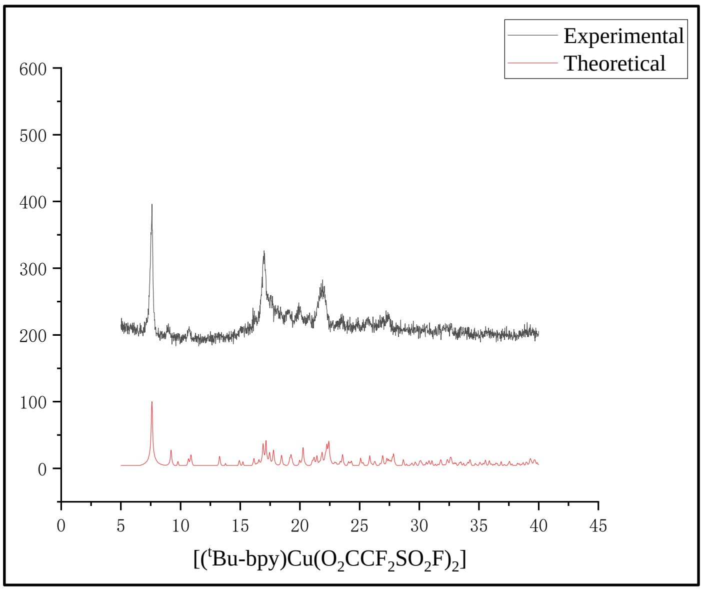

Figure S2 XRD spectrum of [(^{t}Bu-bpy)Cu($O_2CF_2SO_2F$)$_2$]

(b) ESI-MS spectrum of (bpy)Cu($O_2CCF_2SO_2F$)$_2$

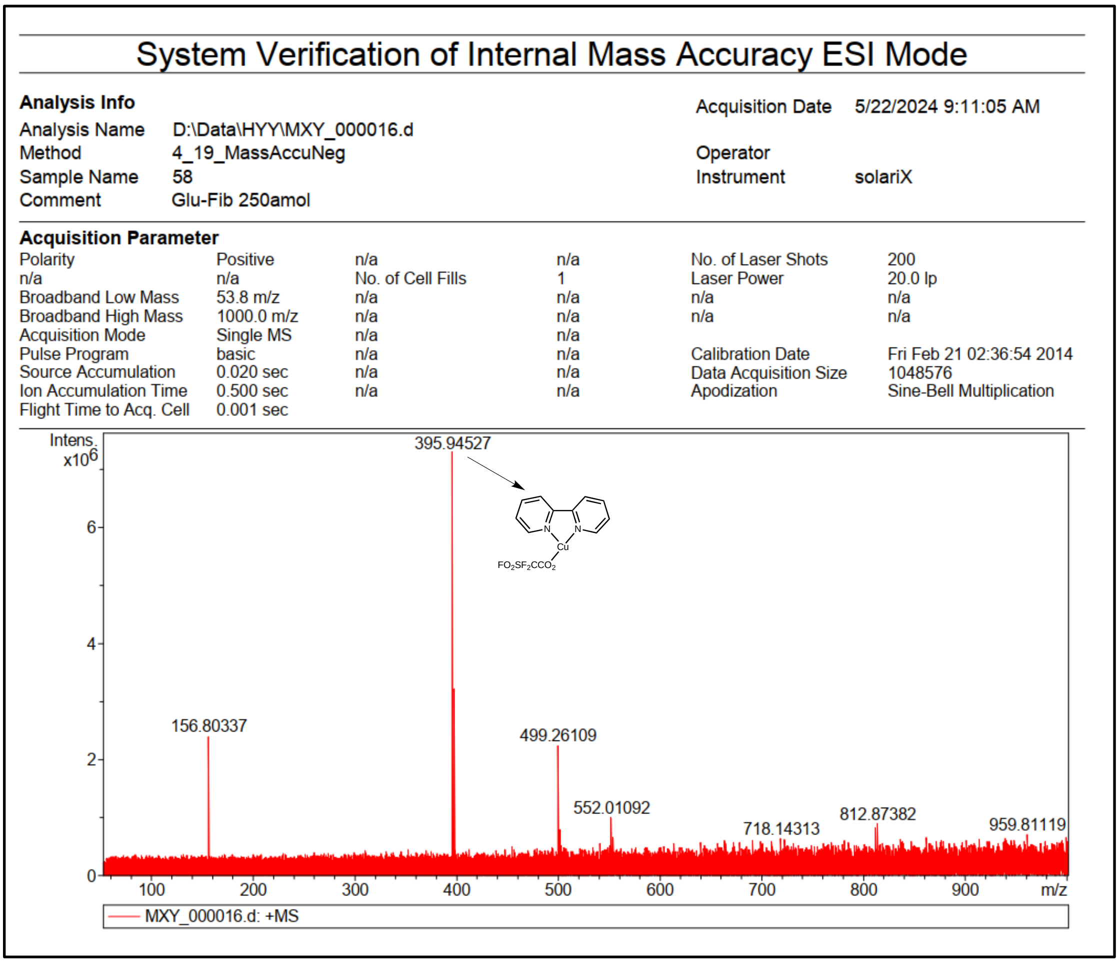

Figure S3 ESI-MS spectrum of [(bpy)Cu($O_2CCF_2SO_2F$)$_2$]

(c) X-ray crystallographic studies of [(^{t}Bu-bpy)Cu($O_2CF_2SO_2F$)$_2$]

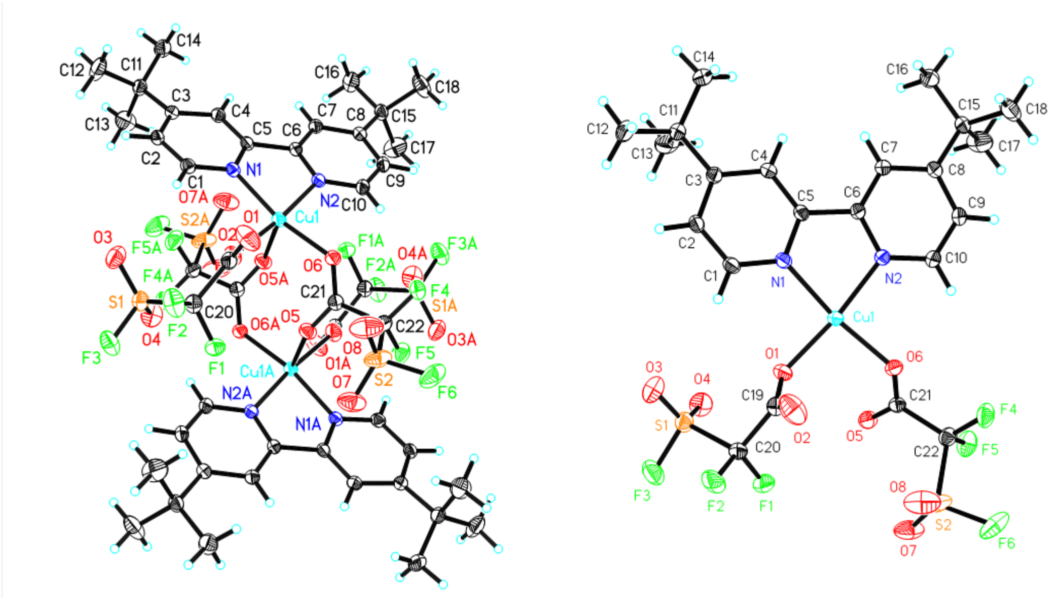

Suitable crystals were mounted on grass fibers or sealed in thin-walled glass capillaries. X-ray intensity data of (^{t}Bu-bpy)Cu($O_2CCF_2SO_2F)_2$ was collected on a Bruker SMART CCD-APEX diffractometer employing graphite monochromated Mo-Kα radiation (λ=0.71073 Å) and using the ω-2θ scan technique. The intensity data were corrected for Lorentz and polarization effects. Refinement was by full-matrix least-squares techniques based on F to minimize the quantity $\sum w(|Fo|-|Fc|)2$ with $w = 1/\sigma 2$ (F). Non-hydrogen atoms were refined anisotropically, and hydrogen atoms were refined isotropically. Crystal data and data collection parameters are summarized in Table S1.

Table S1. Crystal data and structure refinement for [(^{t}Bu-bpy)Cu($O_2CCF_2SO_2F)_2$]

Identification code	mo_d8v21944_0m
Empirical formula	C44 H48 Cu2 F12 N4 O16 S4
Formula weight	1372.18

Temperature	213(2) K	
Wavelength	0.71073 Å	
Crystal system	Triclinic	
Space group	P -1	
Unit cell dimensions	a = 10.3516(18) Å	a= 87.461(5)°.
	b = 11.812(2) Å	b= 70.344(5)°.
	c = 12.354(2) Å	g = 79.786(6)°.
Volume	1399.8(4) Å^3	
Z	1	
Density (calculated)	1.628 Mg/m^3	
Absorption coefficient	1.017 mm^{-1}	
F(000)	698	
Crystal size	0.160 x 0.110 x 0.060 mm^3	
Theta range for data collection	2.507 to 26.000°.	
Index ranges	-12<=h<=12, -14<=k<=14, -14<=l<=15	
Reflections collected	21655	
Independent reflections	5495 [R(int) = 0.0342]	
Completeness to theta = 25.242°	99.7 %	
Absorption correction	Semi-empirical from equivalents	
Max. and min. transmission	0.7456 and 0.5967	

Refinement method	Full-matrix least-squares on F^2
Data / restraints / parameters	5495 / 19 / 394
Goodness-of-fit on F^2	1.076
Final R indices [I>2sigma(I)]	R1 = 0.0344, wR2 = 0.0807
R indices (all data)	R1 = 0.0431, wR2 = 0.0862
Extinction coefficient	n/a
Largest diff. peak and hole	0.336 and -0.374 e.Å^{-3}

III. Screening reaction condictions

Table S2 Screening on the temperature during the ligand addition process[a]

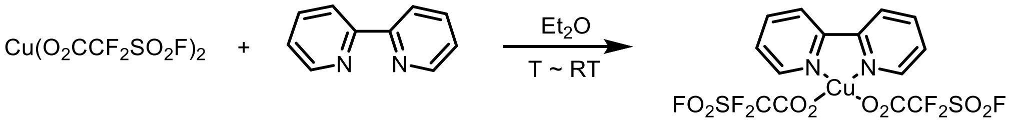

Entry	Equivalent	T (℃)	Yield (%)[b]
1	1:1	-196	85
**2**	**1:1**	**-78**	**97**
3	1:1	-40	96
4	1:1	0	0

[a] Reaction conditions: $Cu(O_2CCF_2SO_2F)_2$ (0.2 mmol, 1.0 eq), bpy (0.2 mmol, 1.0 equiv), redistilled Et_2O (2 mL), Ar atmosphere, 30 min. [b] Yields were determined by ^{19}F NMR with trifluorotoluene as internal standard

Table S3 Screening on the equivalent of $Cu(O_2CCF_2SO_2F)_2$ and ligand[a]

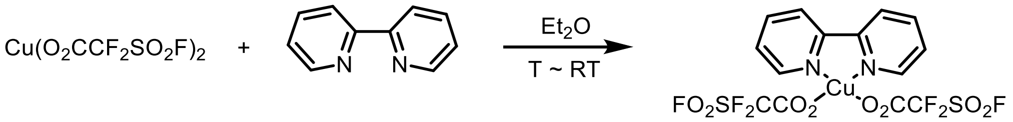

Entry	Equivalent	T (℃)	Yield (%)[b]
1	1:1.2	-78	85
**2**	**1:1**	**-78**	**97**
3	1:0.9	-78	96

4	1:0.8	-78	96

[a] Reaction conditions: $Cu(O_2CCF_2SO_2F)_2$ (0.2 mmol, 1.0 eq), bpy, redistilled Et_2O (2 mL), Ar atmosphere, 30 min. [b] Isolated yields are reported.

Table S4 Screening on the reaction time[a]

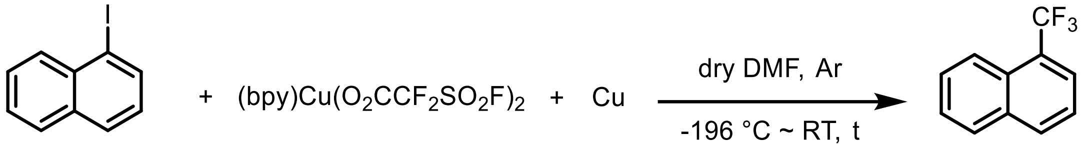

Entry	t (h)	Yield (%)[b]
1	3	23
**2**	**5**	**25**
3	12	23

[a] Reaction conditions: 1-iodonaphthalene (0.2 mmol, 1.0 equiv), $(bpy)Cu(O_2CCF_2SO_2F)_2$ (0.3 mmol, 1.5 equiv), Cu (0.3 mmol, 1.5 equiv), DMF (2 mL), Ar atmosphere. [b] Yields were determined by ^{19}F NMR with trifluorotoluene as internal standard.

Table S5 Screening on the reaction temperature and equivalent of $(bpy)Cu(O_2CCF_2SO_2F)_2$ and Cu[a]

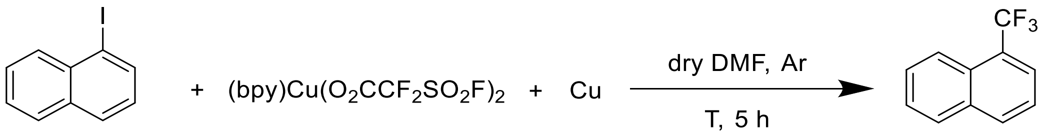

Entry	[Cu] (equiv)	Cu (equiv)	T (°C)	Yield (%)[b]
1	2.0	2.0	-196 ~ RT	62

2	2.0	2.0	RT	53
3	2.0	2.5	RT	52
4	3.0	3.0	RT	50
**5**	**2.0**	**2.0**	**RT ~ 60**	**85**
6	2.0	2.0	RT ~ 80	82

[a] Reaction conditions: 1-iodonaphthalene (0.2 mmol, 1.0 equiv), [(bpy)Cu($O_2CCF_2SO_2F$)$_2$], Cu, DMF (2 mL), Ar atmosphere. [b] Yields were determined by ^{19}F NMR with trifluorotoluene as internal standard.

Table S6 Screening on solvent[a]

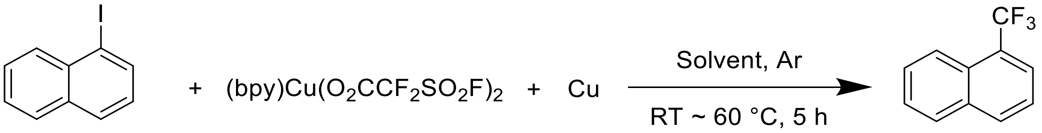

Entry	Solvent	Yield (%)[b]
1	MeCN	0
2	DMF	85
**3**	**NMP**	**89**

[a] Reaction conditions: 1-iodonaphthalene (0.2 mmol, 1.0 equiv), [(bpy)Cu($O_2CCF_2SO_2F$)$_2$] (0.4 mmol, 2.0 equiv), Cu (0.4 mmol, 2.0 equiv), solvent, Ar atmosphere. [b] Yields were determined by ^{19}F NMR with trifluorotoluene as internal standard.

IV. Analytical data for compounds 4a–4x

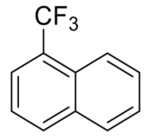

1-(trifluoromethyl)naphthalene (4a). Obtained as colorless oil in 68% yield (53 mg).^{1}**H NMR** (400 MHz, $CDCl_3$): δ 8.25 (d, *J* = 8.3 Hz, 1H), 8.03 (d, *J* = 8.4 Hz, 1H), 7.92 (dd, *J* = 13.8, 7.5 Hz, 2H), 7.71-7.56 (m, 2H), 7.51 (t, *J* = 7.8 Hz, 1H).; ^{19}F NMR (376 MHz, $CDCl_3$): δ -59.71 (s, 3F); ^{13}C NMR (101 MHz, $CDCl_3$): δ 134.05, 132.88, 129.13, 128.90, 127.77, 126.73, 126.34 (q, *J* =10.0 Hz), 124.93 (q, *J* = 274.6 Hz), 124.41 (q, *J* = 2.4 Hz), 124.28, 123.57 ppm. HRMS (EI) m/z: $[M]^+$ Calcd for $C_{11}H_7F_3$ 196.0496; Found 196.0500. The analytical data are consistent with literature values[1].

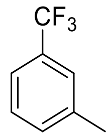

1-methyl-3-(trifluoromethyl)benzene (4b). 53% ^{19}F NMR yield. Characterization of **4b** in the reaction solution: Crude ^{19}F NMR (unlocked): δ −62.58 (s, 3F) ppm. GC-MS (EI): m/z = 160.1 (M^+). The analytical data are consistent with literature values[2].

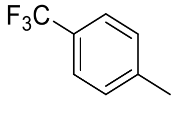

1-methyl-4-(trifluoromethyl)benzene (4c). 46% ^{19}F NMR yield. Characterization of **4c** in the reaction solution: Crude ^{19}F NMR (unlocked): δ

−61.94 (s, 3F) ppm. GC-MS (EI): m/z = 160.1 (M^+). The analytical data are consistent with literature values[2].

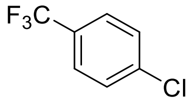

1-chloro-4-(trifluoromethyl)benzene (4d). 55% ^{19}F NMR yield. Characterization of **4d** in the reaction solution: Crude ^{19}F NMR (unlocked): δ −62.27 (s, 3F) ppm. GC-MS (EI): m/z = 180.0 (M^+). The analytical data are consistent with literature values[2].

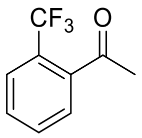

1-(2-(trifluoromethyl)phenyl)ethan-1-one (4e). Obtained as colorless oil in 82% yield (62 mg). ^{1}H NMR (400 MHz, $CDCl_3$): δ 7.67 (d, *J* = 7.2 Hz, 1H), 7.61-7.47 (m, 2H), 7.44 (d, *J* = 8.4 Hz, 1H), 2.54 (s, 3H); ^{19}F NMR (376 MHz, $CDCl_3$): δ -58.26 (s, 3F); ^{13}C NMR (101 MHz, $CDCl_3$): δ = 201.89, 140.46 (q, *J* = 2.1 Hz), 131.99, 130.21, 127.12, 126.70 (q, *J* = 5.1 Hz), 125.07, 123.71 (q, *J* = 274.6 Hz), 30.54 (d, *J* = 1.8 Hz) ppm. HRMS (EI) m/z: $[M]^+$ Calcd for $C_9H_7F_3O$ 188.0439; Found 188.0409. The analytical data are consistent with literature values[3].

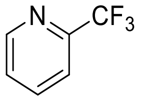

2-(trifluoromethyl)pyridine (4f). 80% ^{19}F NMR yield. Characterization of **4f** in the reaction solution: Crude ^{19}F NMR (unlocked): δ −67.82 (s, 3F) ppm. GC-MS (EI): m/z = 147.0 (M^+). The analytical data are consistent with literature values[2].

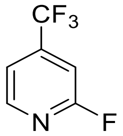

2-fluoro-4-(trifluoromethyl)pyridine (4g). 82% ^{19}F NMR yield. Characterization of **4g** in the reaction solution: Crude ^{19}F NMR (unlocked): δ −60.24 (s, 3F), −61.10 (s, 1F) ppm. GC-MS (EI): m/z = 165.1 (M^+). The analytical data are consistent with literature values[2].

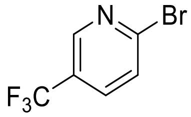

2-bromo-5-(trifluoromethyl)pyridine (4h). Obtained as white solid in 30% yield (27 mg). ^{1}H NMR (400 MHz, $CDCl_3$): δ 8.66 (s, 1H), 7.78(d, *J* = 8.4 Hz, 1H), 7.65 (d, *J* = 8.4 Hz, 1H); ^{19}F NMR (376 MHz, $CDCl_3$): δ -62.49 (s, 3F); ^{13}C NMR (101 MHz, $CDCl_3$): δ 147.38, 146.18, 135.51, 128.49, 126.38, 123.3 (q, *J* = 273.7 Hz) ppm. HRMS (EI) m/z: $[M]^+$ Calcd for $C_6H_3BrF_3N$ 224.9400; Found 224.9401. The analytical data are consistent with literature values[4].

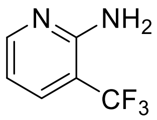

3-(trifluoromethyl)pyridin-2-amine (4i). Obtained as a yellow solid in 8% yield (5 mg). ^{1}H NMR (400 MHz, $CDCl_3$): δ 7.93 (d, *J* = 6.8 Hz, 1H), 7.83 (d, *J* = 6.8 Hz, 1H), 6.68 (t, *J* = 6.8 Hz, 1H), 6.00 (br, 2H); ^{19}F NMR (376 MHz, $CDCl_3$): δ

-65.20 (s, 3F); ^{13}C NMR (101 MHz, $CDCl_3$): δ 158.29, 142.73 (q, *J* = 4.8), 136.24 (t, *J* = 4.3 Hz), 124.44 (d, *J* = 272.4 Hz), 120.38, 109.88 ppm. HRMS (ESI) m/z: $[M]^+$ Calcd for $C_6H_5F_3N_2$ 163.0478; Found 162.0405. The analytical data are consistent with literature values[5].

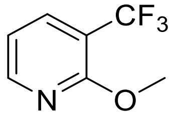

2-methoxy-3-(trifluoromethyl)pyridine (4j). Obtained as a yellow liquid in 60% yield (43 mg). ^1H NMR (400 MHz, $CDCl_3$): δ 8.32 (d, *J* = 4.4 Hz, 1H), 7.85 (d, *J* = 7.2 Hz, 1H), 6.96 (t, *J* = 6.8 Hz, 1H), 4.04 (s, 3H); ^{19}F NMR (376 MHz, $CDCl_3$): δ -63.99 (s, 3F); ^{13}C NMR (101 MHz, $CDCl_3$): δ 161.0, 157.6, 136.5 (q, *J* = 4.8 Hz), 123.1 (d, *J* = 270 Hz), 116.0, 113.4 (d, *J* = 32.8 Hz), 54.2 ppm. HRMS (EI) m/z: $[M]^+$ Calcd for $C_7H_6F_3NO$ 177.0393; Found 177.0401.

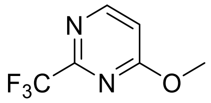

4-methoxy-2-(trifluoromethyl)pyrimidine (4k). Obtained as a yellow liquid in 80% yield (57 mg). ^1H NMR (400 MHz, $CDCl_3$): δ 8.53 (d, *J* = 6.0 Hz, 1H), 6.87 (d, *J* = 5.6 Hz, 1H), 4.04 (s, 3H); ^{19}F NMR (376 MHz, $CDCl_3$): δ -71.07 (s, 3F); ^{13}C NMR (101 MHz, $CDCl_3$): δ 170.4, 157.5, 156.5 (d, *J* = 36.6 Hz), 119.4 (q, *J* = 274.0 Hz), 110.7, 54.6 ppm. HRMS (EI) m/z: $[M]^+$ Calcd for $C_6H_5F_3N_2O$ 178.0345; Found 178.0354. The analytical data are consistent with literature values[2].

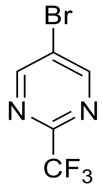

5-bromo-2-(trifluoromethyl)pyrimidine (4l). Obtained as a yellow solid in 35% yield (32 mg). ^1H NMR (400 MHz, $CDCl_3$): δ 8.97 (s, 2H); ^{19}F NMR (376 MHz, $CDCl_3$): δ -70.10 (s, 3F); ^{13}C NMR (101 MHz, $CDCl_3$): δ 158.94, 155.06 (d, *J* = 38.0 Hz), 123.17, 118.19 (q, *J* = 276.0 Hz) ppm. HRMS (EI) m/z: $[M]^+$ Calcd for $C_5H_2BrF_3N_2$ 225.9354; Found 225.9353. The analytical data are consistent with literature values[6].

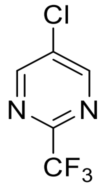

5-chloro-2-(trifluoromethyl)pyrimidine (4m). Obtained as a yellow solid in 90% yield (66 mg). ^1H NMR (400 MHz, $CDCl_3$): δ 8.86 (s, 2H); ^{19}F NMR (376 MHz, $CDCl_3$): δ -70.00 (s, 3F); ^{13}C NMR (101 MHz, $CDCl_3$): δ 156.71, 154.66 (d, *J* = 37.6 Hz), 134.10, 119.43 (q, *J* = 275.2 Hz) ppm. HRMS (EI) m/z: $[M]^+$ Calcd for $C_5H_2ClF_3N_2$ 181.9856; Found 181.9859. The analytical data are consistent with literature values[2].

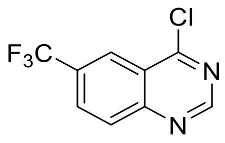

4-chloro-6-(trifluoromethyl)quinazoline (4n). Obtained as white solid in 31% yield (29 mg). ^1H NMR (400 MHz, $CDCl_3$): δ 9.17 (s, 1H), 8.59 (s, 1H), 8.23 (d, *J* = 8.8 Hz, 1H), 8.15 (d, *J* = 8.8 Hz, 1H); ^{19}F NMR (376 MHz, $CDCl_3$): δ -62.76 (s, 3F); ^{13}C NMR (101 MHz, $CDCl_3$): δ 163.52, 155.50, 154.03, 152.29, 143.83, 134.60,

130.71, 130.39, 123.17 (q, J = 272.9 Hz) ppm. HRMS (EI) m/z: $[M]^+$ Calcd for $C_9H_4ClF_3N_2$ 232.0014; Found 232.0015.

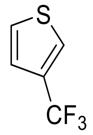

3-(trifluoromethyl)thiophene (4o). 55% ^{19}F NMR yield. Characterization of **4o** in the reaction solution: Crude ^{19}F NMR (unlocked): δ −54.07 (s, 3F) ppm. GC-MS (EI): m/z = 152.0 (M^+). The analytical data are consistent with literature values[2].

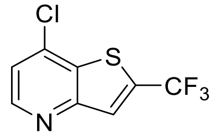

7-chloro-2-(trifluoromethyl)thieno[3,2-b]pyridine (4p). Obtained as white solid in 55% yield (52 mg). ^1H NMR (400 MHz, $CDCl_3$): δ 8.70 (d, J = 4.8 Hz, 1H), 7.94 (d, J = 0.3 Hz, 1H), 7.42 (d, J = 4.8 Hz, 1H); ^{19}F NMR (376 MHz, $CDCl_3$): δ -57.33 (s, 3F); ^{13}C NMR (101 MHz, $CDCl_3$): δ 155.28, 149.52, 138.69, 136.35 (d, J = 39.0 Hz), 134.16, 127.47 (q, J = 4.0 Hz), 122.04 (d, J = 276.9 Hz), 120.73 ppm. HRMS (EI) m/z: $[M]^+$ Calcd for $C_8H_3ClF_3NS$ 236.9618; Found 236.9627.

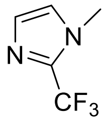

1-methyl-2-(trifluoromethyl)-1H-imidazole (4q). 37% ^{19}F NMR yield. Characterization of **4q** in the reaction solution: Crude ^{19}F NMR (unlocked): δ −60.96 (s, 3F) ppm. GC-MS (EI): m/z = 150.1 (M^+). The analytical data are consistent with literature values[1].

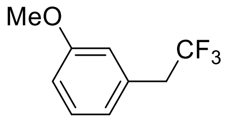

1- methoxy-3-(2,2,2-trifluoroethyl)benzene (4t). Obtained as white solid in 38% yield (29 mg). ^{1}H NMR (400 MHz, $CDCl_3$): δ 7.29 (t, *J* = 8.0 Hz, 1H), 6.93-6.87 (m, 2H), 6.85 (s, 1H), 3.82 (s, 1H), 3.35 (q, *J* = 10.8 Hz, 2H); ^{19}F NMR (376 MHz, $CDCl_3$): δ -65.82 (t, *J* = 10.5 Hz, 3F); ^{13}C NMR (101 MHz, $CDCl_3$): δ 159.9, 131.7(q, *J* = 2.8 Hz), 129.8, 125.9(d, *J* = 275.3 Hz), 122.6, 116.1, 113.6, 55.4, 40.4(q, *J* = 29.6 Hz) ppm. HRMS (EI) m/z: $[M]^+$ Calcd for $C_9H_9F_3O$ 190.0603; Found 190.0605.

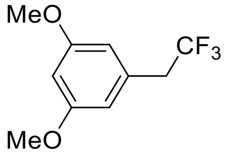

1,3-dimethoxy-5-(2,2,2-trifluoroethyl)benzene (4u). Obtained as white solid in 41% yield (45 mg). ^{1}H NMR (400 MHz, $CDCl_3$): δ 6.54 (d, *J* = 2.4 Hz, 2H), 6.40 (t, *J* = 2Hz, 1H), 4.43 (s, 2H), 3.79 (s, 6H); ^{19}F NMR (376 MHz, $CDCl_3$): δ -65.61 (t, *J* = 10.5 Hz, 3F); ^{13}C NMR (101 MHz, $CDCl_3$): δ 160.96, 132.19, 125.80 (q, *J* = 276.8 Hz), 108.36, 99.96, 55.30, 40.38 (q, *J* = 29.7 Hz) ppm. HRMS (EI) m/z: $[M]^+$ Calcd for $C_{10}H_{11}F_3O_2$ 220.0703; Found 220.0711. The analytical data are consistent with literature values[7].

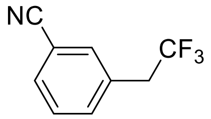

3-(2,2,2-trifluoroethyl)benzonitrile (4v). Obtained as white solid in 20% yield (15 mg). ^{1}H NMR (400 MHz, $CDCl_3$): δ 7.67 (s, 1H), 7.62 (d, *J* = 7.6 Hz, 1H), 7.57 (d, *J* = 8.0 Hz, 1H), 7.46 (t, *J* = 7.6 Hz, 1H), 4.46 (s, 2H); ^{19}F NMR (376 MHz, $CDCl_3$):

δ -65.76 (t, *J* = 10.5 Hz); ^{13}C NMR (101 MHz, $CDCl_3$): δ 134.60, 133.66, 131.96, 131.72, 129.66, 125.20 (q, *J* = 277.7 Hz), 118.18, 113.16, 39.85 (q, *J* = 30.3 Hz) ppm. HRMS (EI) m/z: $[M]^+$ Calcd for $C_9H_6F_3N$ 185.0451; Found 185.0452. The analytical data are consistent with literature values[8].

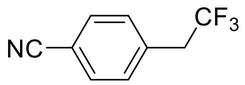

4-(2,2,2-trifluoroethyl)benzonitrile (4w). Obtained as white solid in 36% yield (27 mg). ^{1}H NMR (400 MHz, $CDCl_3$): δ 7.66 (d, *J* = 8.0 Hz, 2H), 7.43 (d, *J* = 8.0 Hz, 2H), 3.44 (q, *J* = 10.8 Hz, 2H); ^{19}F NMR (376 MHz, $CDCl_3$): δ -65.55 (t, *J* = 10.5 Hz, 3F); ^{13}C NMR (101 MHz, $CDCl_3$): δ 135.44 (d, *J* = 3.0 Hz), 132.58, 131.09, 125.27 (q, *J* = 277.0 Hz), 118.44, 112.53, 40.37 (q, *J* = 30.2 Hz) ppm. HRMS (EI) m/z: $[M]^+$ Calcd for $C_9H_6F_3N$ 185.0448; Found 185.0452. The analytical data are consistent with literature values[2].

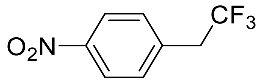

1-nitro-4-(2,2,2-trifluoroethyl)benzene (4x). Obtained as white solid in 21% yield (17 mg). ^{1}H NMR (400 MHz, $CDCl_3$): δ 8.21 (d, *J* = 8.8 Hz, 2H), 7.47 (d, *J* = 8.4 Hz, 2H), 3.5 (q, *J* = 10.4 Hz, 2H); ^{19}F NMR (376 MHz, $CDCl_3$): δ -65.46 (t, *J* = 10.5Hz, 3F); ^{13}C NMR (101 MHz, $CDCl_3$): δ 148.08, 137.36 (d, *J* = 2.9 Hz), 131.30, 129.89, 125.22 (q, *J* = 277.0 Hz), 40.12 (q, *J* = 30.4 Hz) ppm. HRMS (EI) m/z: $[M]^+$ Calcd for $C_8H_6F_3NO_2$ 205.0350; Found 205.0351. The analytical data are consistent with literature values[2].

V. References

1. Chen, M.; Buchwald, S. L. Rapid and Efficient Trifluoromethylation of Aromatic and Heteroaromatic Compounds Using Potassium Trifluoroacetate Enabled by a Flow System. *Angew. Chem., Int. Ed.* **2013**, *52*, 11628-11631.
2. Zhao, G.; Wu, H.; Xiao, Z.; Chen, Q.-Y.; Liu, C. Trifluoromethylation of haloarenes with a new trifluoro-methylating reagent $Cu(O_2CCF_2SO_2F)_2$. *RSC Adv.* **2016**, *6*, 50250-50254.
3. Li, T., Hammond, G.B., and Xu, B. (2021). Cobalt-Catalyzed Aerobic Oxidative Cleavage of Alkyl Aldehydes: Synthesis of Ketones, Esters, Amides, and α-Ketoamides. *Chem. Eur. J.* **2021**, *27*, 9737-9741.
4. Ouyang, Y.; Xu, X.; Qing, F., Oxidative Coupling Reactions of Arylboronic Acids and Fluoroform-Derived $AgCF_3$. *Chin. J. Org. Chem.* **2020**, *40*, 3426-3430.
5. Dunn, A. D., Nucleophilic displacement in 2-chloro(trifluoromethyl)pyridines with amines and ammonia. *J. Fluorine Chem.* **1999**, *93*, 153-157.
6. Aikawa, K.; Nakamura, Y.; Yokota, Y.; Toya, W.; Mikami, K. Stable but Reactive Perfluoroalkylzinc Reagents: Application in Ligand-free Copper-catalyzed Perfluoroalkylation of Aryl Iodides. *Chem. Eur. J.* **2015**, *21*, 96-100.

7. Kawai, H., Furukawa, T., Nomura, Y., Tokunaga, E., and Shibata, N. Cu-Mediated Chemoselective Trifluoromethylation of Benzyl Bromides Using Shelf-Stable Electrophilic Trifluoromethylating Reagents. *Org. Lett.* **2011**, *13*, 14, 3596–3599.

8. Kautzky, J. A.; Wang, T.; Evans, R. W.; MacMillan, D. W. C., Decarboxylative Trifluoromethylation of Aliphatic Carboxylic Acids. *J. Am. Chem. Soc.* **2018**, *140*, 6522-6526.

VI. Copies of ^{1}H NMR, ^{19}F NMR and ^{13}C NMR spectra

^{1}H NMR spectrum of 1-(trifluoromethyl)naphthalene (4a) (400 MHz, $CDCl_3$)

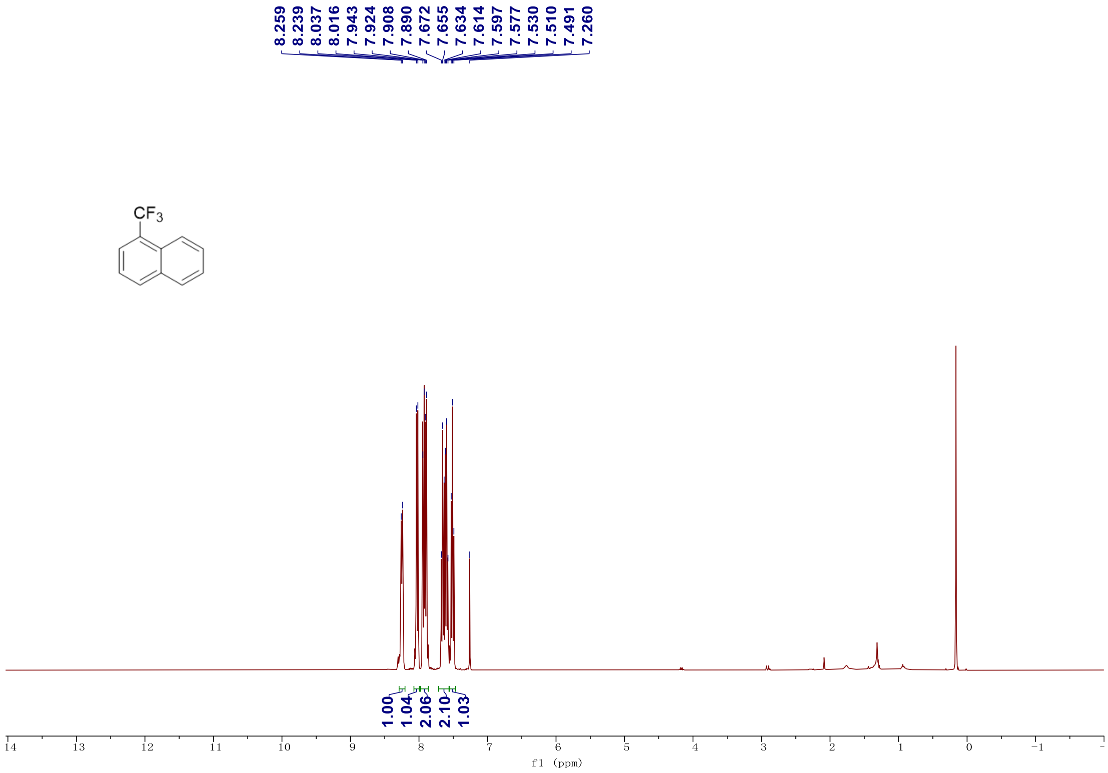

^{19}F NMR spectrum of 1-(trifluoromethyl)naphthalene (4a) (376 MHz, $CDCl_3$)

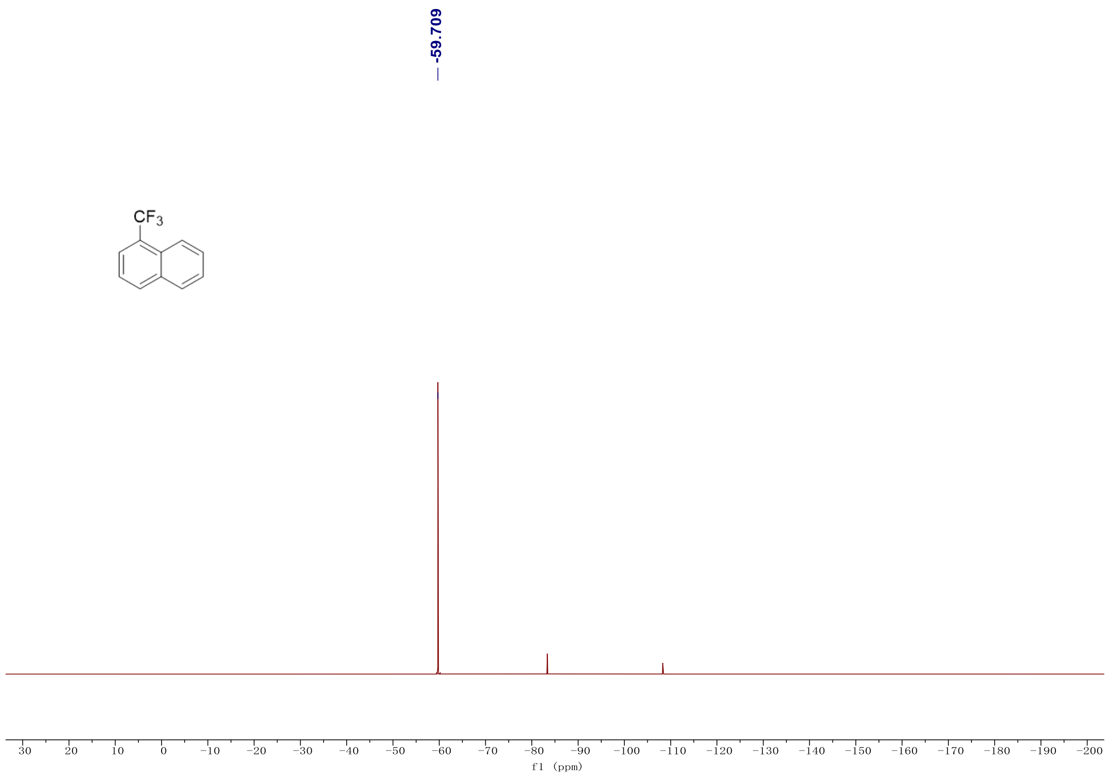

^{13}C NMR spectrum of 1-(trifluoromethyl)naphthalene (4a) (101 MHz, $CDCl_3$)

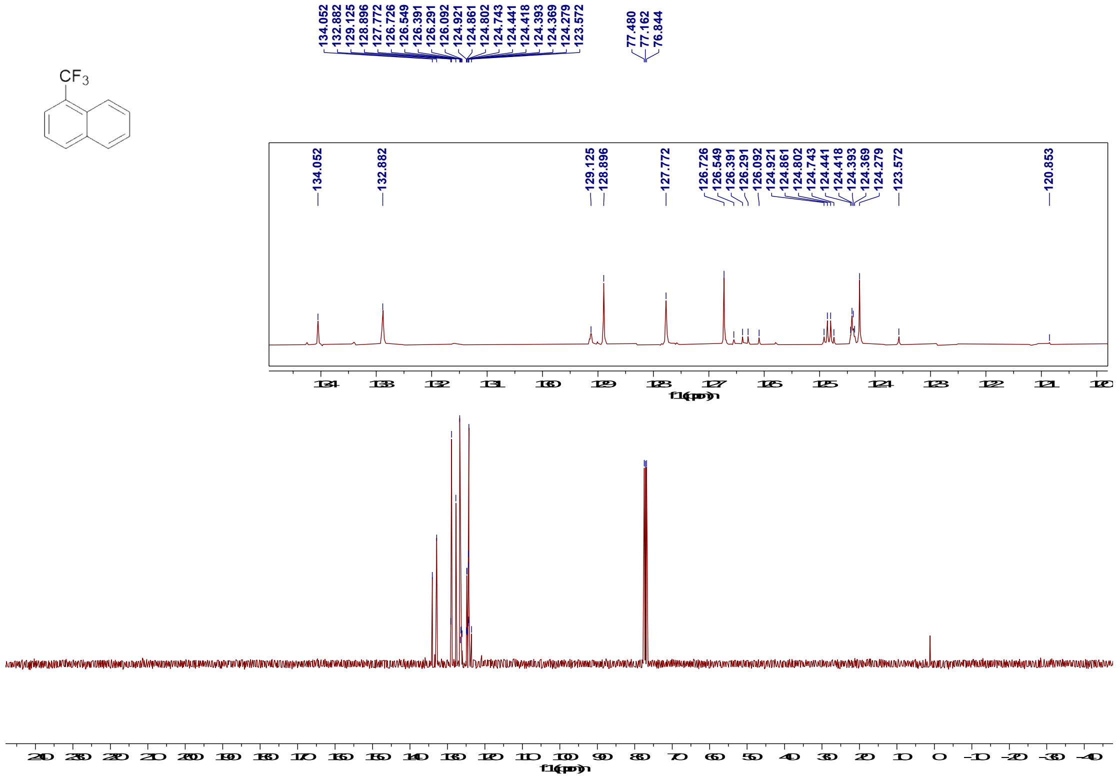

^{1}H NMR spectrum of 1-(2-(trifluoromethyl)phenyl)ethan-1-one (4e) (400 MHz, $CDCl_3$)

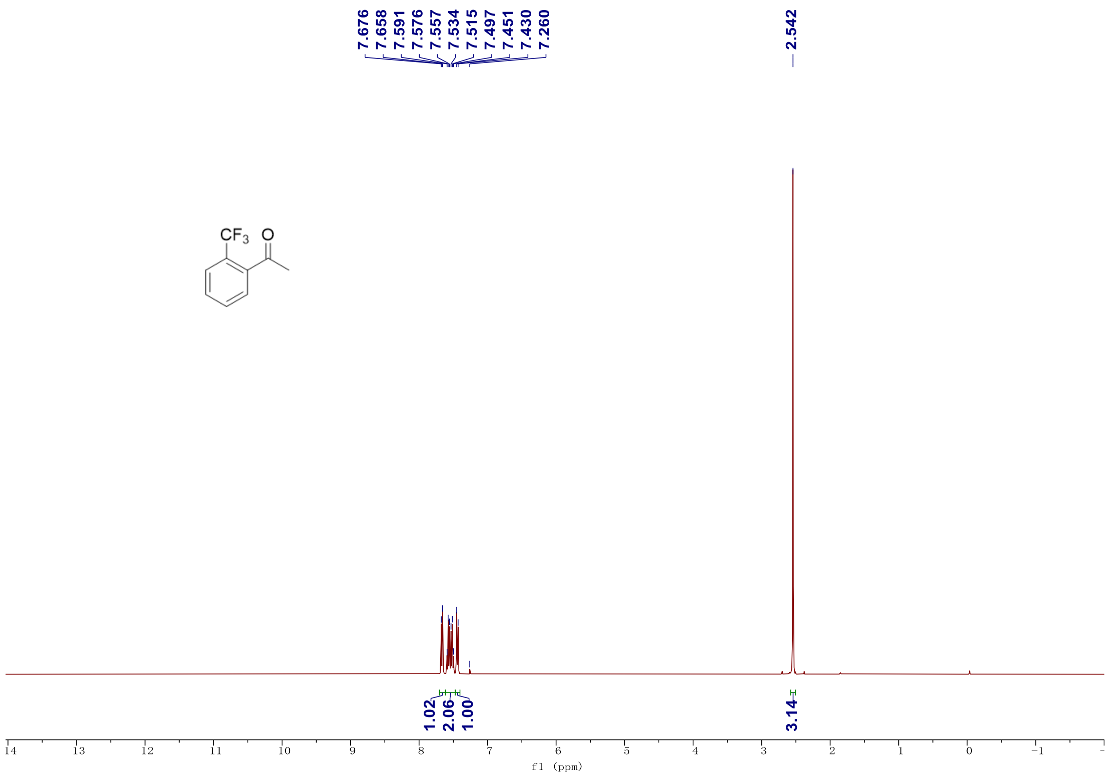

^{19}F NMR spectrum of 1-(2-(trifluoromethyl)phenyl)ethan-1-one (4e) (376 MHz, $CDCl_3$)

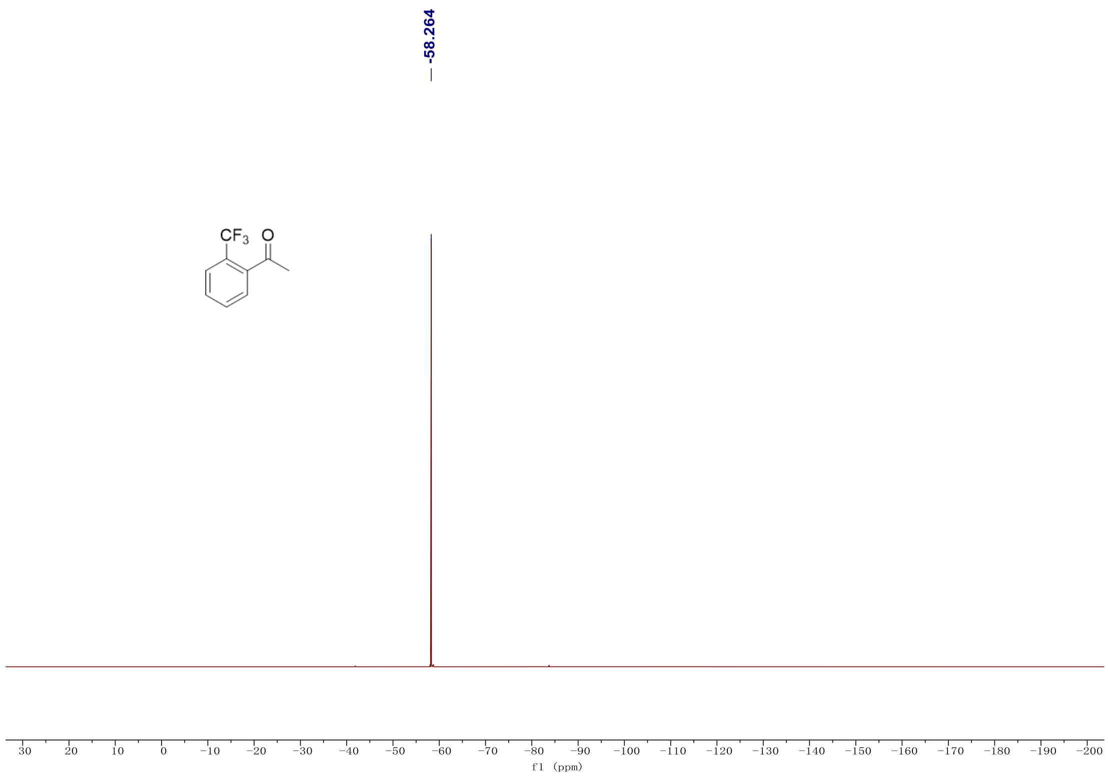

^{13}C NMR spectrum of 1-(2-(trifluoromethyl)phenyl)ethan-1-one (4e) (101 MHz, $CDCl_3$)

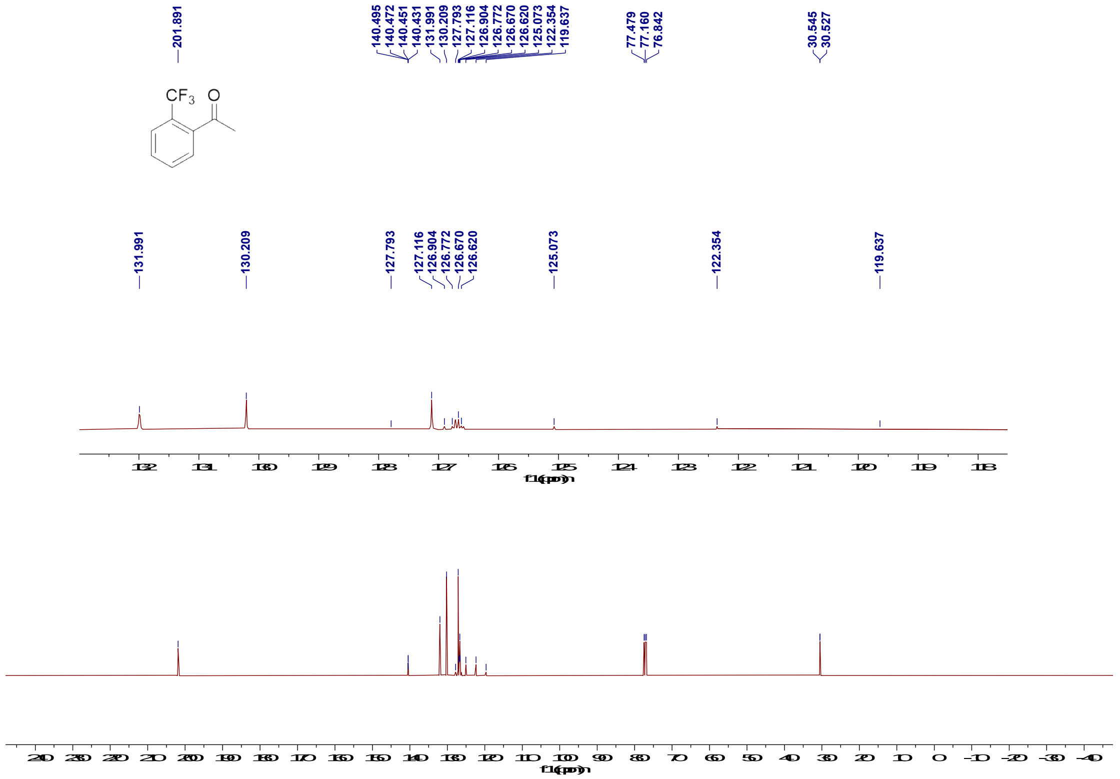

^{1}H NMR spectrum of 2-bromo-5-(trifluoromethyl)pyridine (4h) (400 MHz, $CDCl_3$)

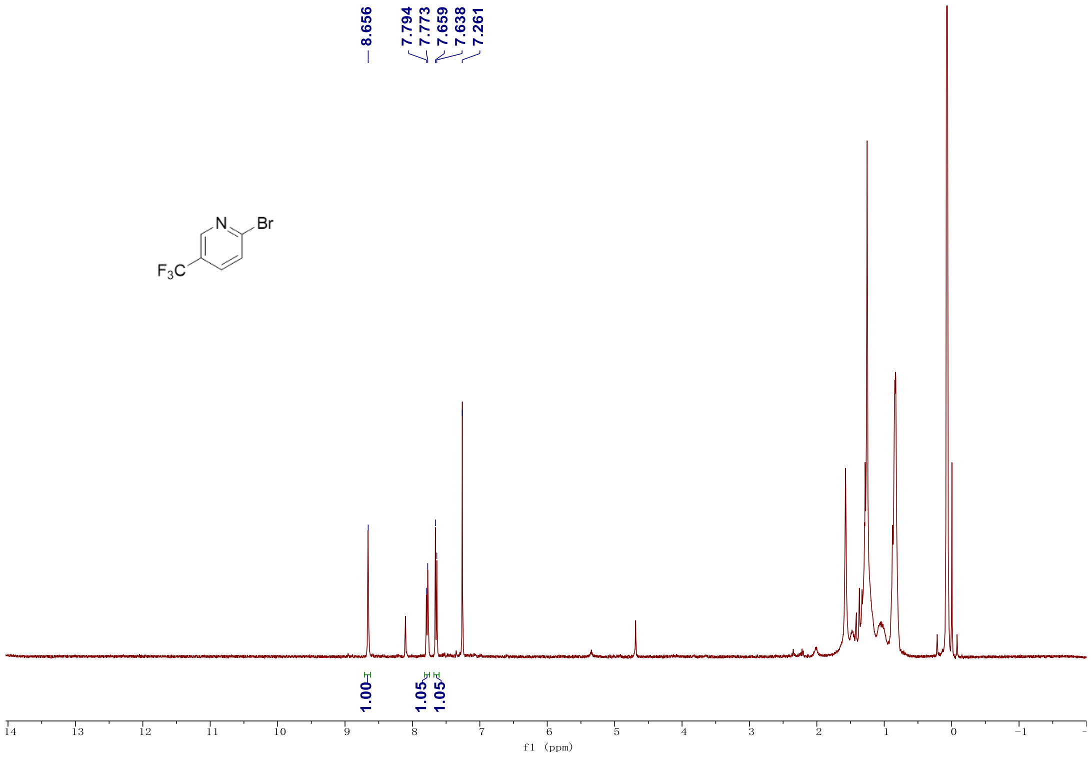

^{19}F NMR spectrum of 2-bromo-5-(trifluoromethyl)pyridine (4h) (376 MHz, $CDCl_3$)

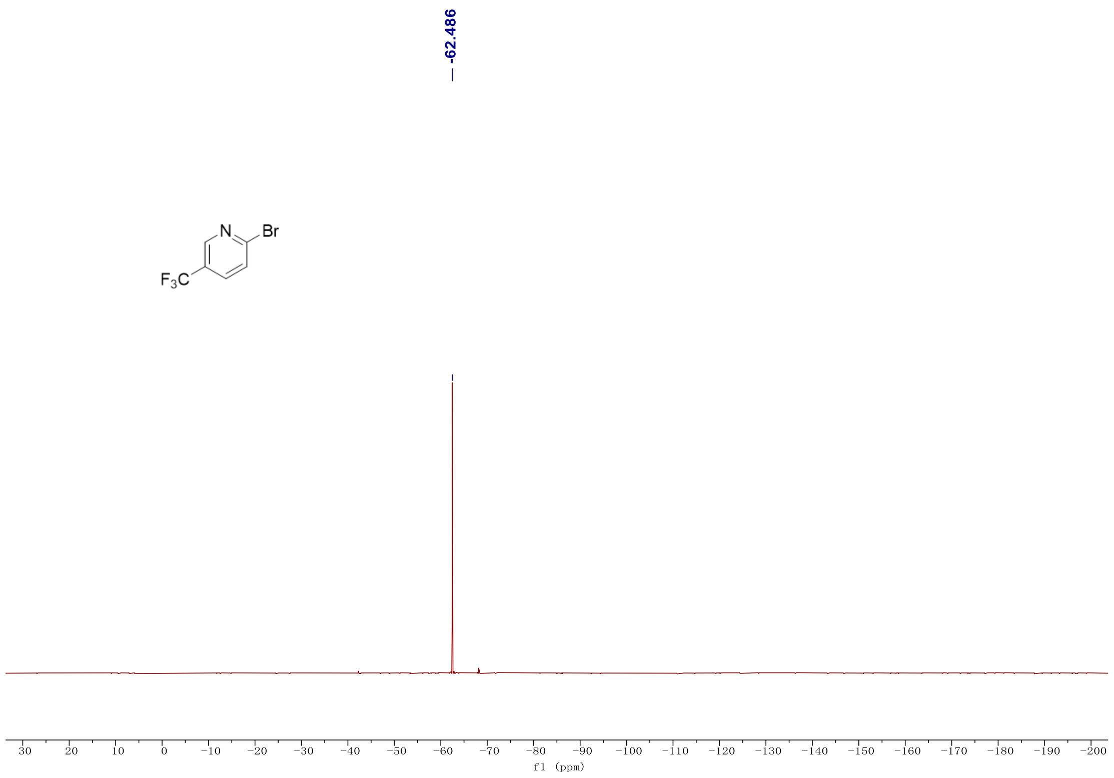

^{13}C NMR spectrum of 2-bromo-5-(trifluoromethyl)pyridine (4h) (101 MHz, $CDCl_3$)

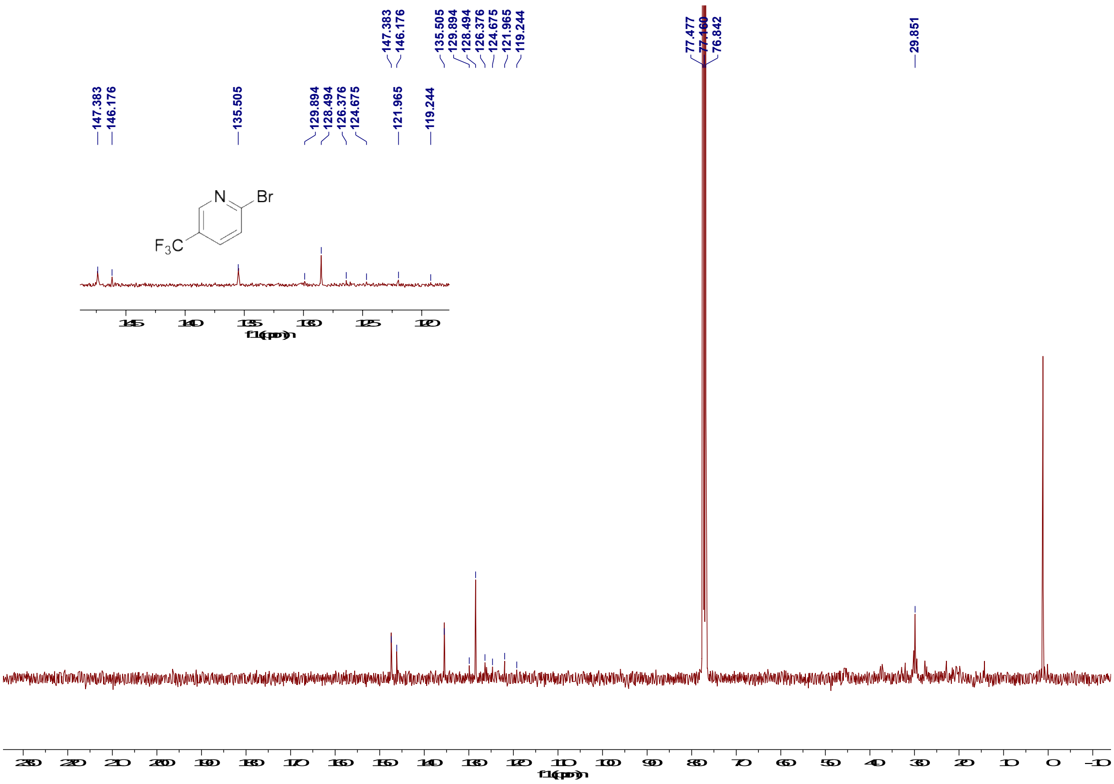

^{1}H NMR spectrum of 3-(trifluoromethyl)pyridin-2-amine (4i) (400 MHz, $CDCl_3$)

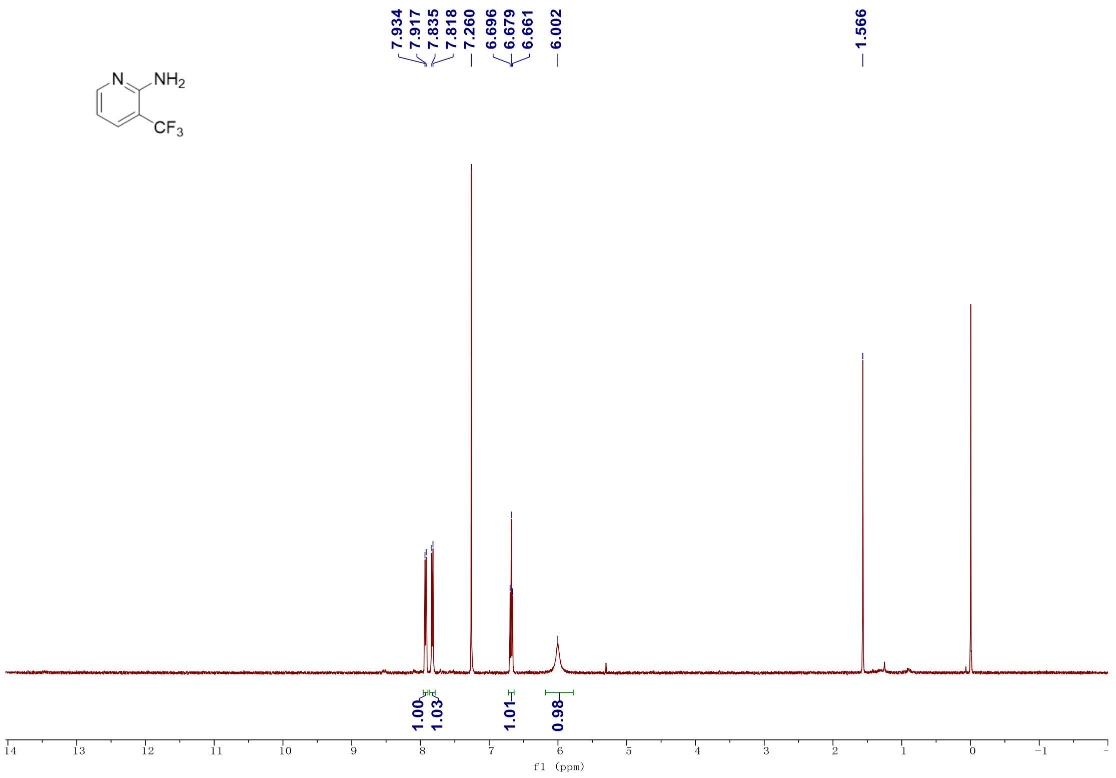

N
NH2
CF3
7.934
7.917
7.835
7.818
7.260
6.696
6.679
6.661
6.002
1.566
1.00
1.03
1.01
0.98
14
13
12
11
10
9
8
7
6
5
4
3
2
1
0
-1
f1 (ppm)

^{19}F NMR spectrum of 3-(trifluoromethyl)pyridin-2-amine (4i) (376 MHz, $CDCl_3$)

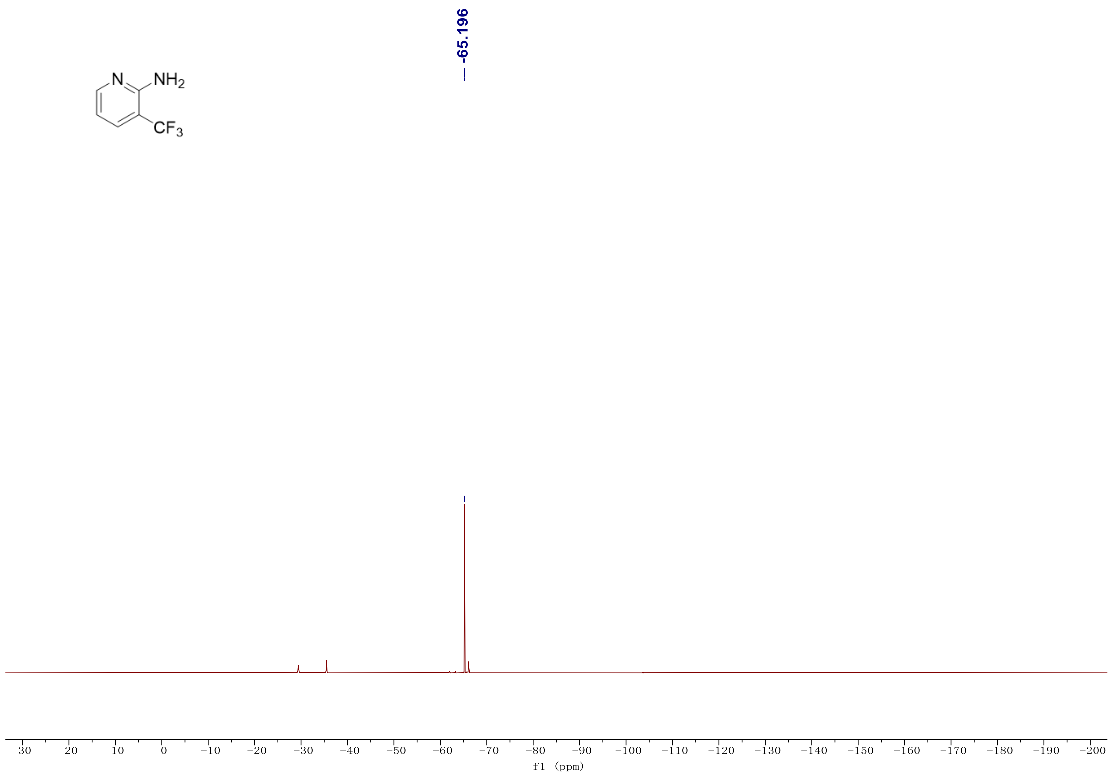

^{13}C NMR spectrum of 3-(trifluoromethyl)pyridin-2-amine (4i) (101 MHz, $CDCl_3$)

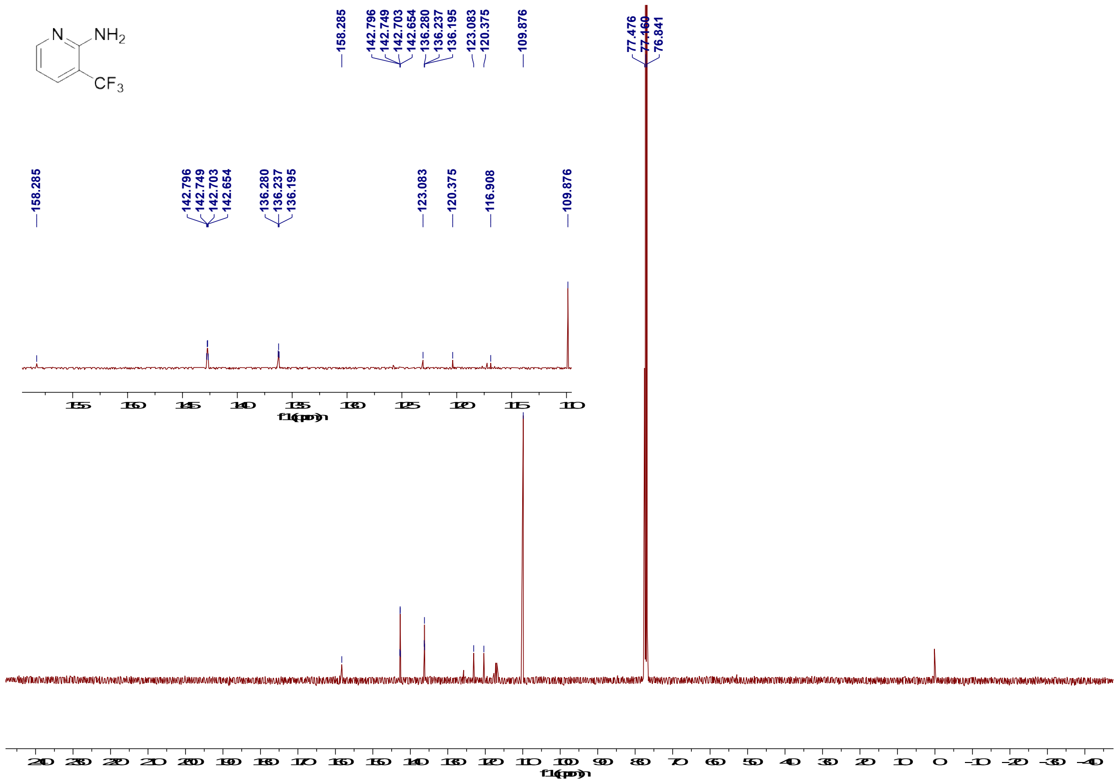

N
NH2
CF3
158.285
142.796
142.749
142.703
142.654
136.280
136.237
136.195
123.083
120.375
109.876
77.476
77.160
76.841
116.908
f1 (ppm)
f1 (ppm)

^{1}H NMR spectrum of 2-methoxy-3-(trifluoromethyl)pyridine (4j) (400 MHz, $CDCl_3$)

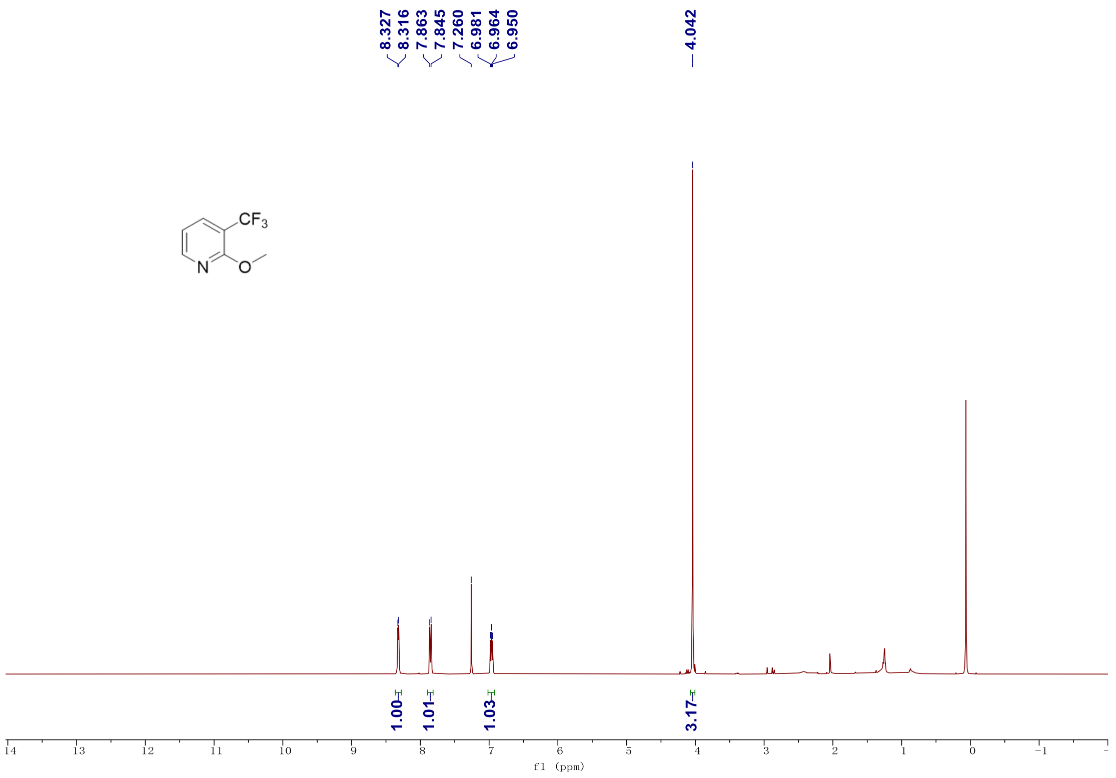

^{19}F NMR spectrum of 2-methoxy-3-(trifluoromethyl)pyridine (4j) (376 MHz, $CDCl_3$)

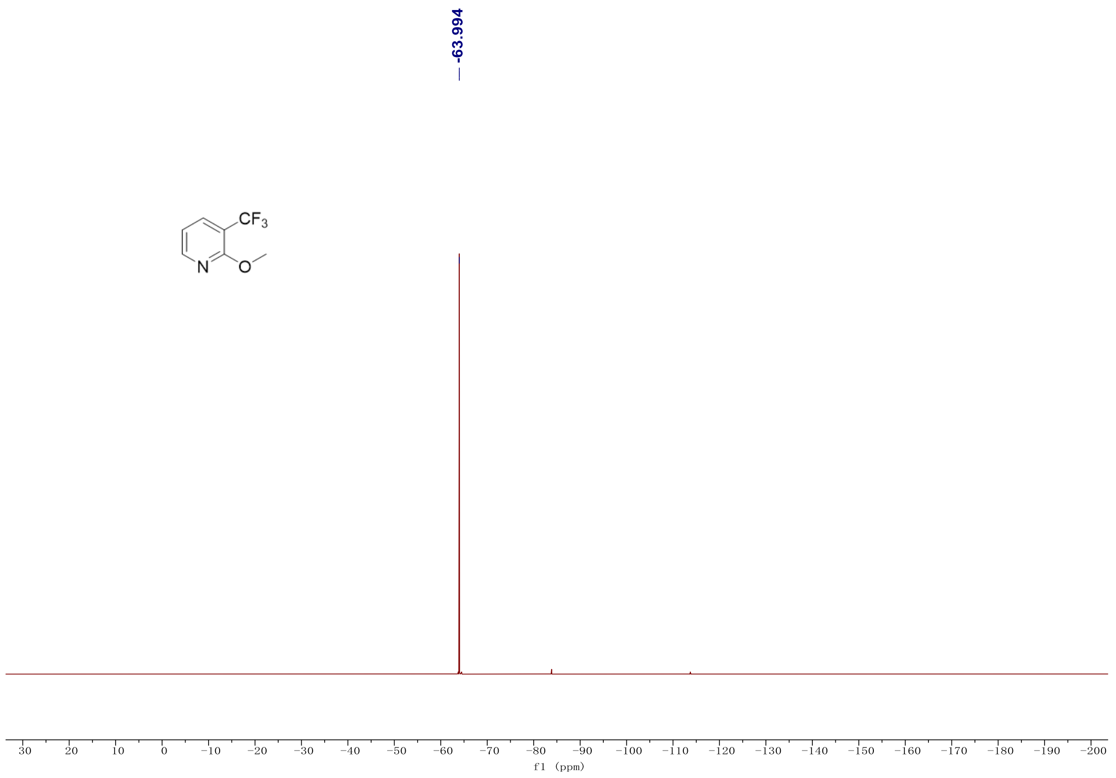
-63.994
CF3
N
O
30 20 10 0 −10 −20 −30 −40 −50 −60 −70 −80 −90 −100 −110 −120 −130 −140 −150 −160 −170 −180 −190 −200
f1 (ppm)

^{13}C NMR spectrum of 2-methoxy-3-(trifluoromethyl)pyridine (4j) (101 MHz, CDCl3)

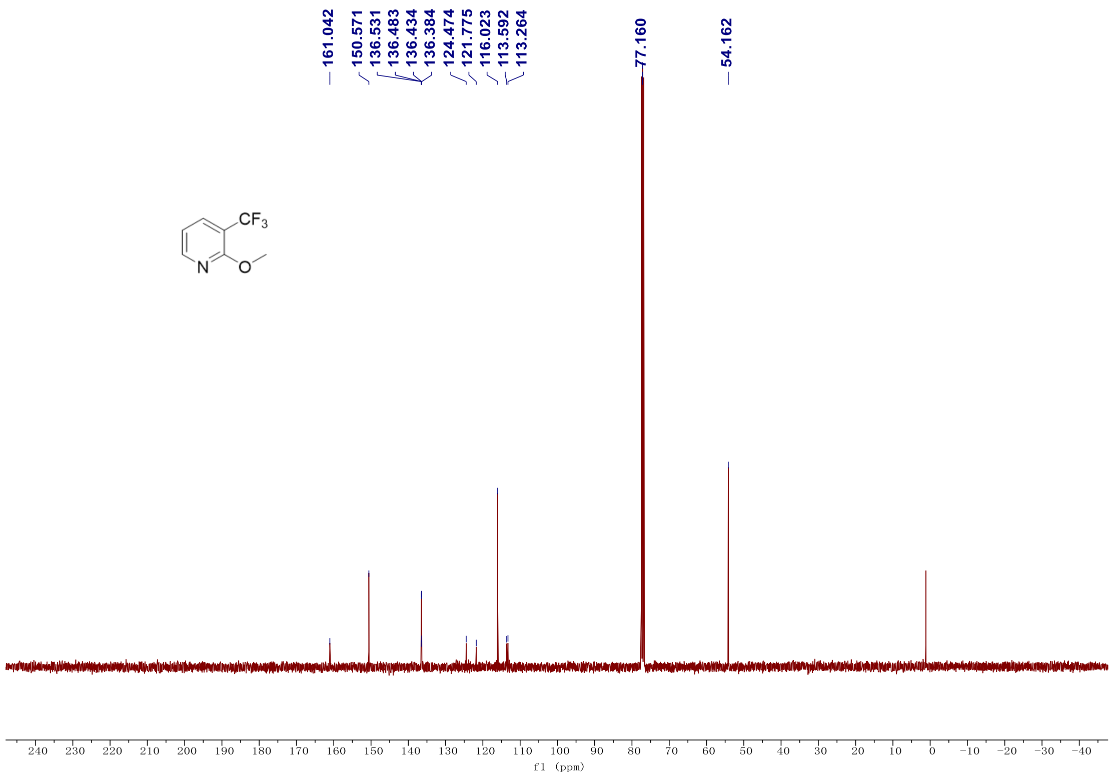

^{1}H NMR spectrum of 4-methoxy-2-(trifluoromethyl)pyrimidine (4k) (400 MHz, CDCl3)

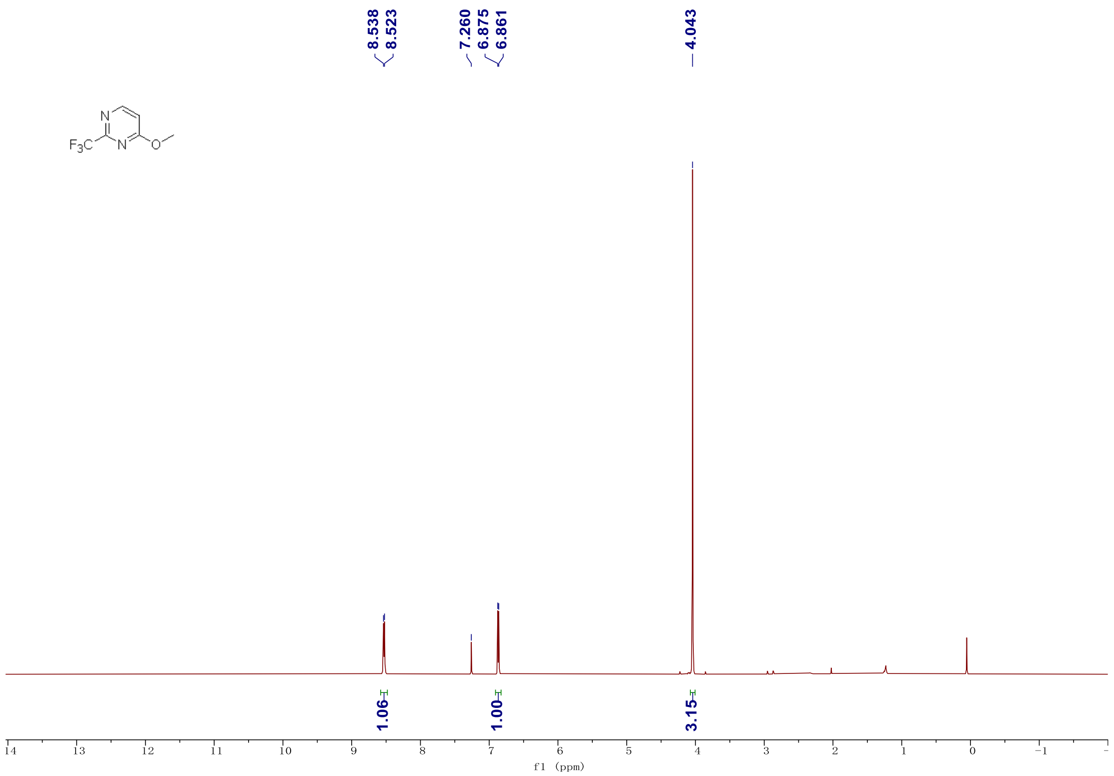

^{19}F NMR spectrum of 4-methoxy-2-(trifluoromethyl)pyrimidine (4k) (376 MHz, $CDCl_3$)

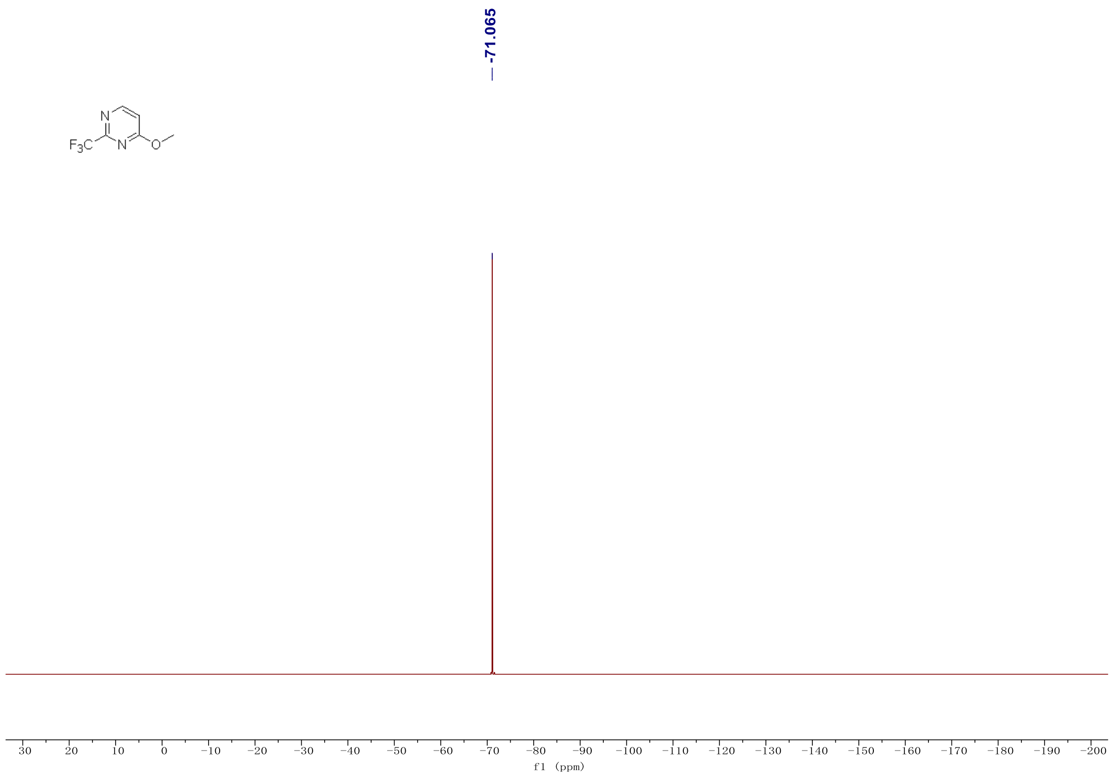

^{13}C NMR spectrum of 4-methoxy-2-(trifluoromethyl)pyrimidine (4k) (101 MHz, $CDCl_3$)

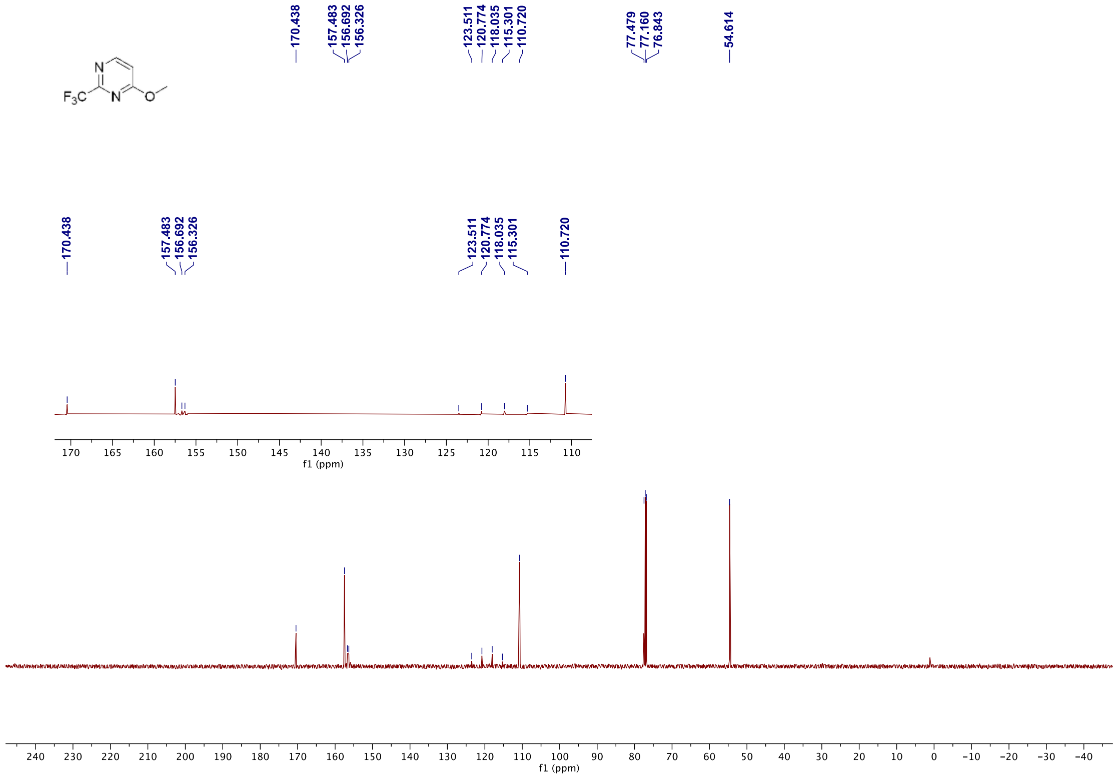

^{1}H NMR spectrum of 5-bromo-2-(trifluoromethyl)pyrimidine (4l) (400 MHz, CDCl$_3$)

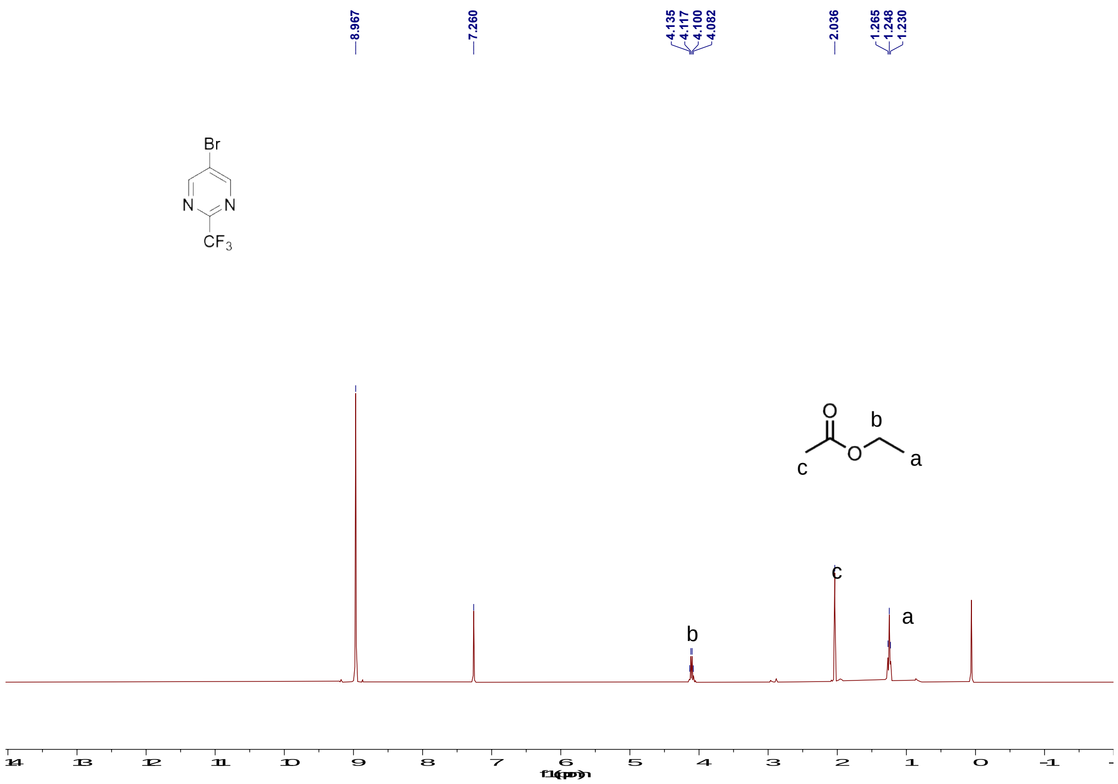

^{19}F NMR spectrum of 5-bromo-2-(trifluoromethyl)pyrimidine (4l) (376 MHz, CDCl$_3$)

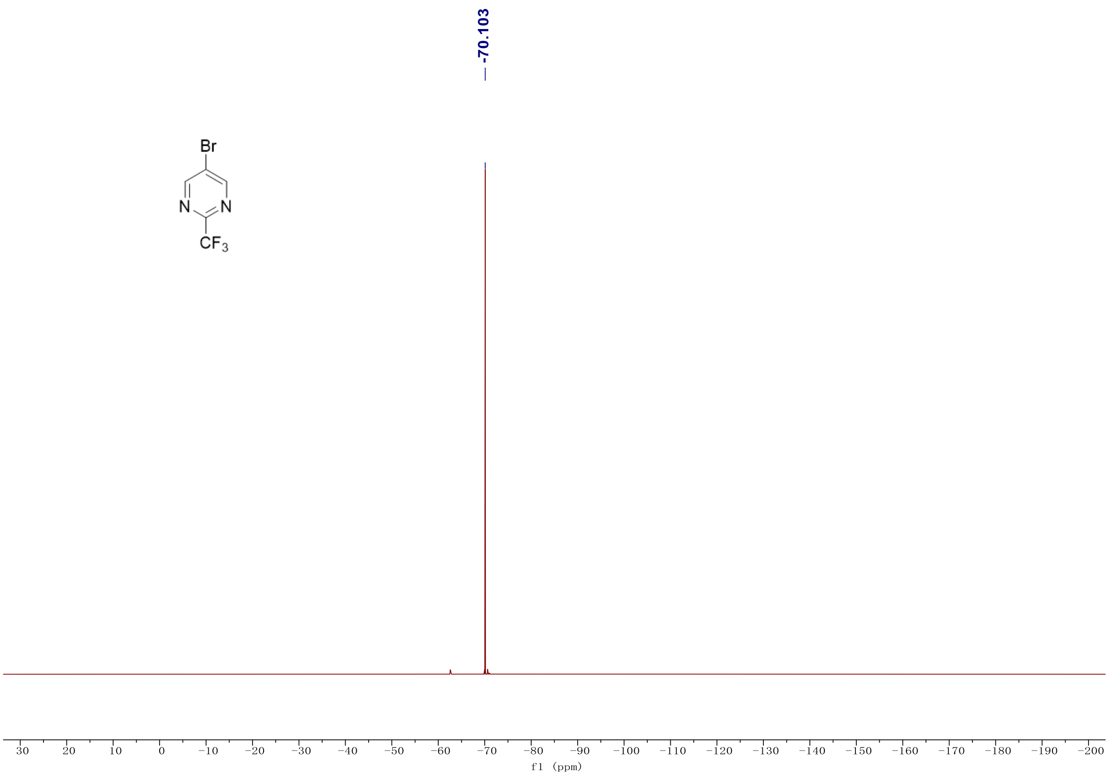

^{13}C NMR spectrum of 5-bromo-2-(trifluoromethyl)pyrimidine (4l) (101 MHz, CDCl₃)

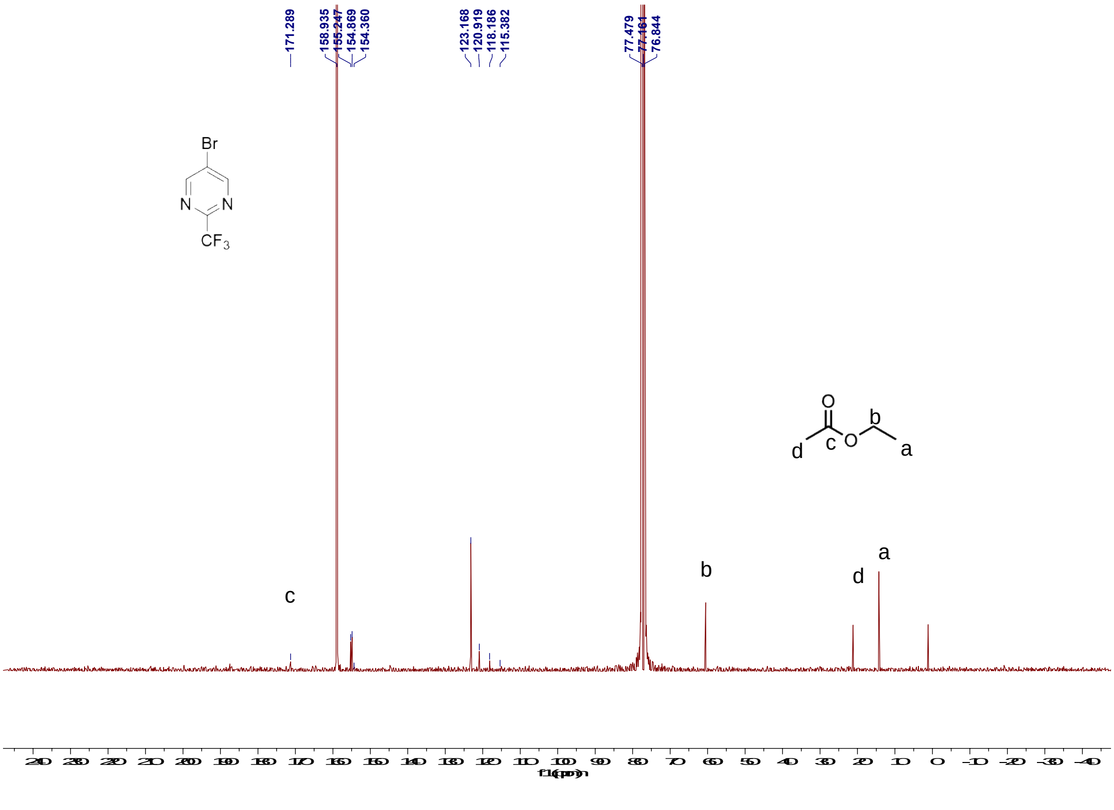

^{1}H NMR spectrum of 5-chloro-2-(trifluoromethyl)pyrimidine (4m) (400 MHz, $CDCl_3$)

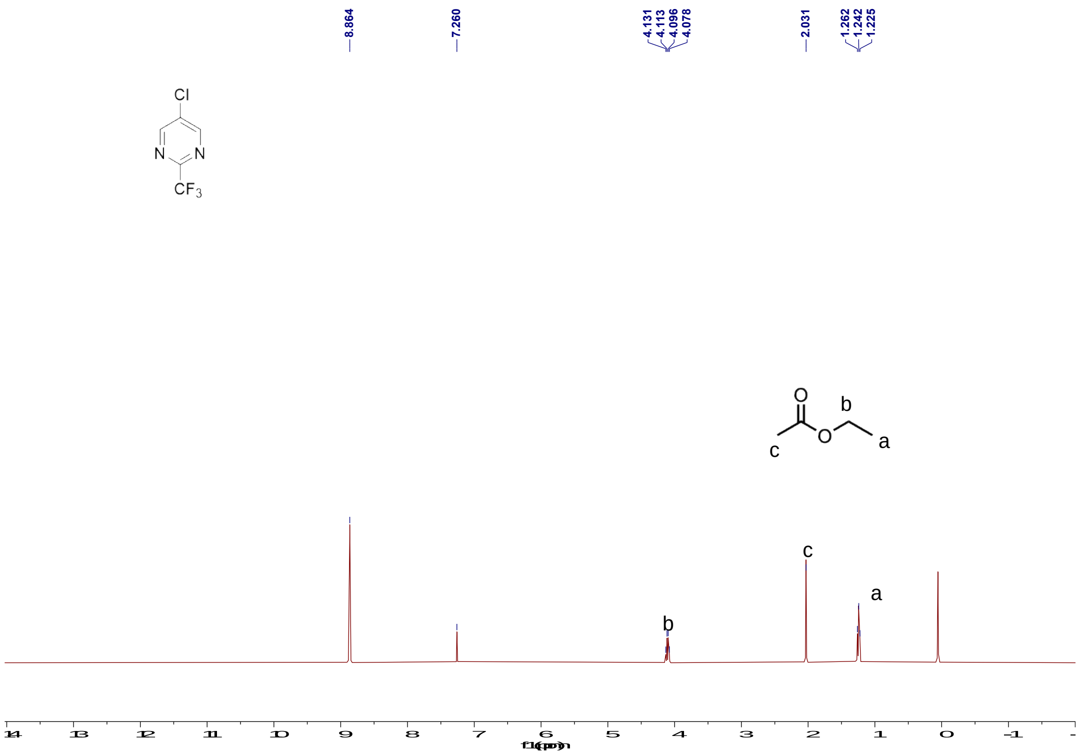

^{19}F NMR spectrum of 5-chloro-2-(trifluoromethyl)pyrimidine (4m) (376 MHz, $CDCl_3$)

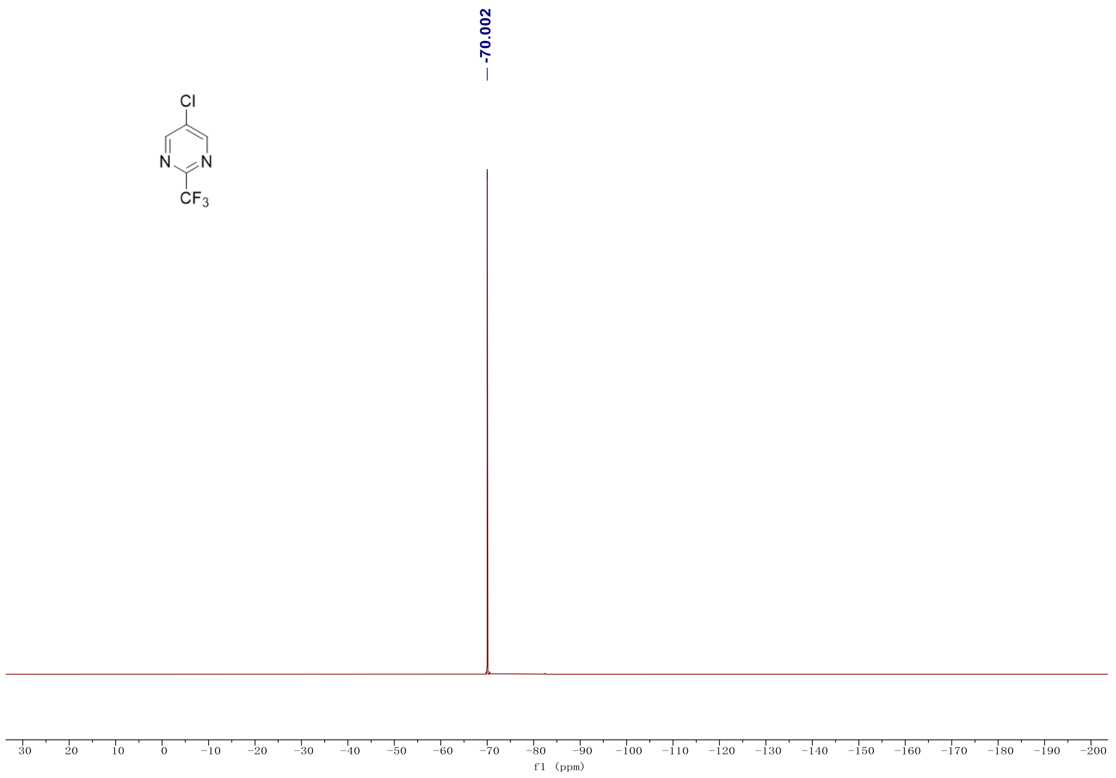

^{13}C NMR spectrum of 5-chloro-2-(trifluoromethyl)pyrimidine (4m) (101 MHz, $CDCl_3$)

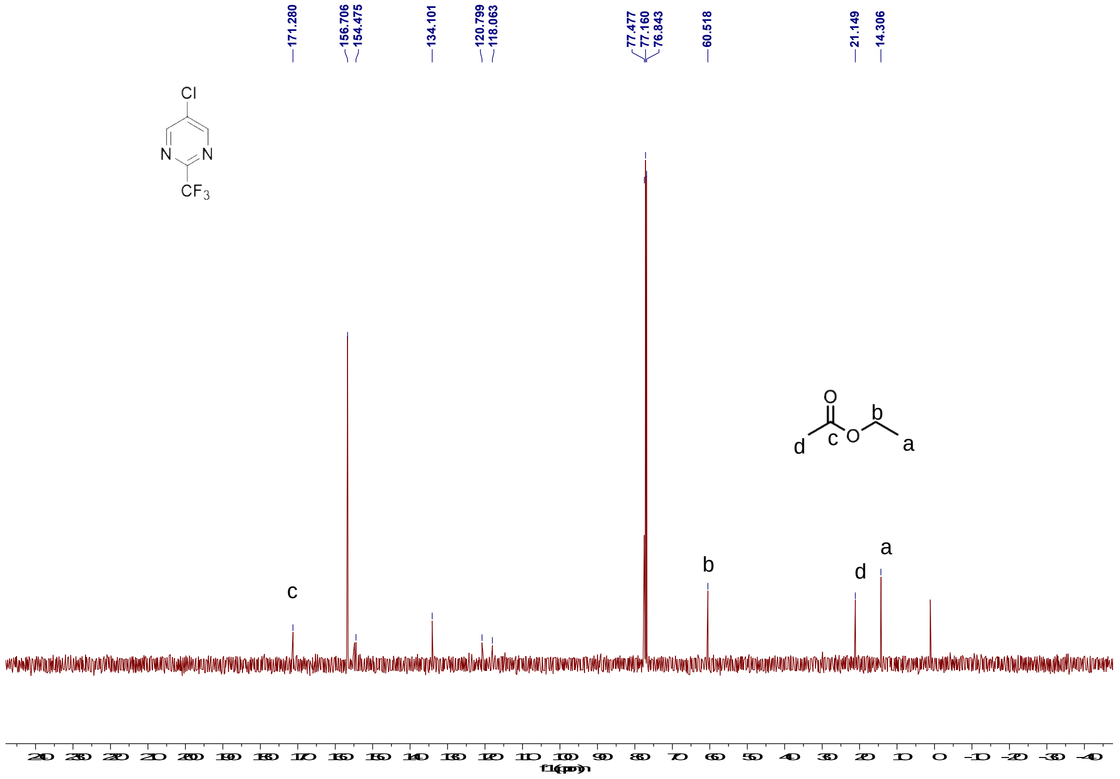

^{1}H NMR spectrum of 4-chloro-6-(trifluoromethyl)quinazoline (4n) (400 MHz, $CDCl_3$)

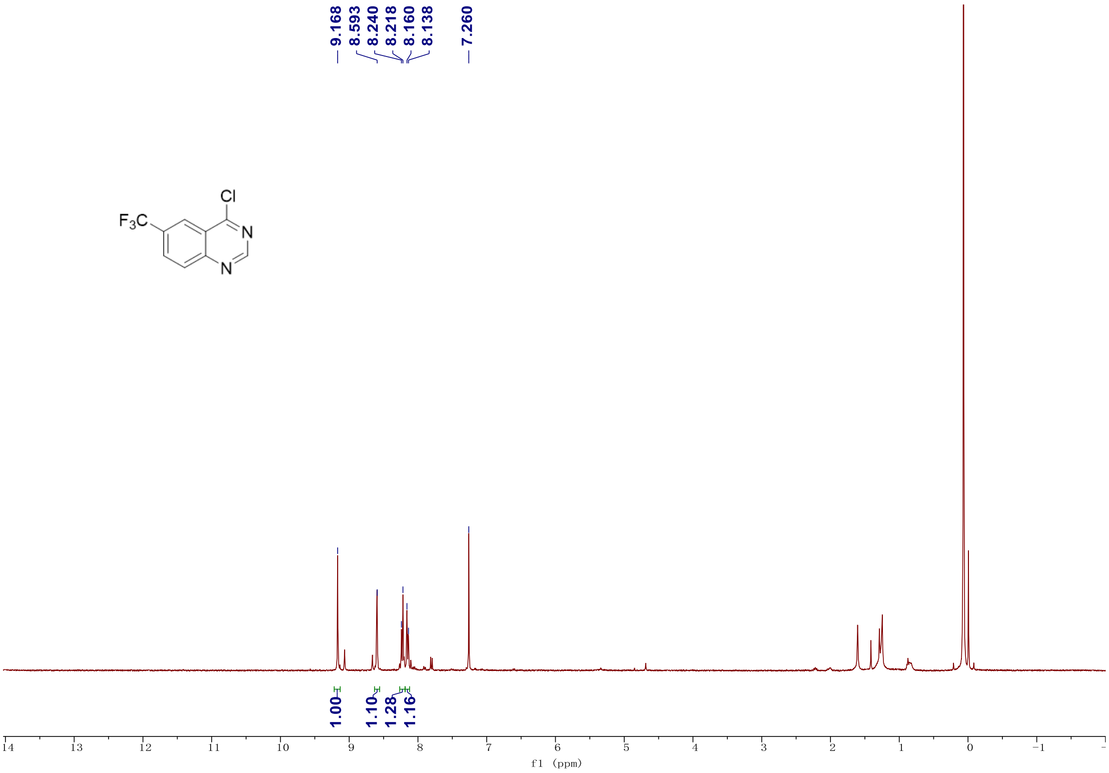

^{19}F NMR spectrum of 4-chloro-6-(trifluoromethyl)quinazoline (4n) (376 MHz, $CDCl_3$)

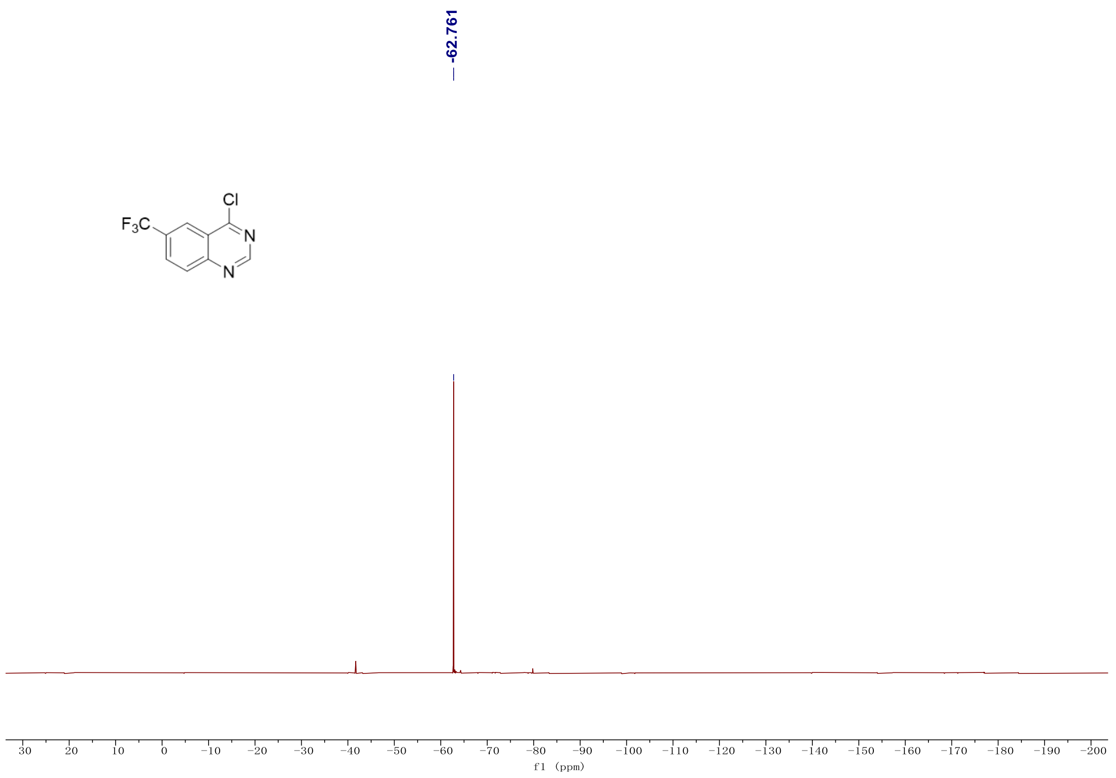

^{13}C NMR spectrum of 4-chloro-6-(trifluoromethyl)quinazoline (4n) (101 MHz, $CDCl_3$)

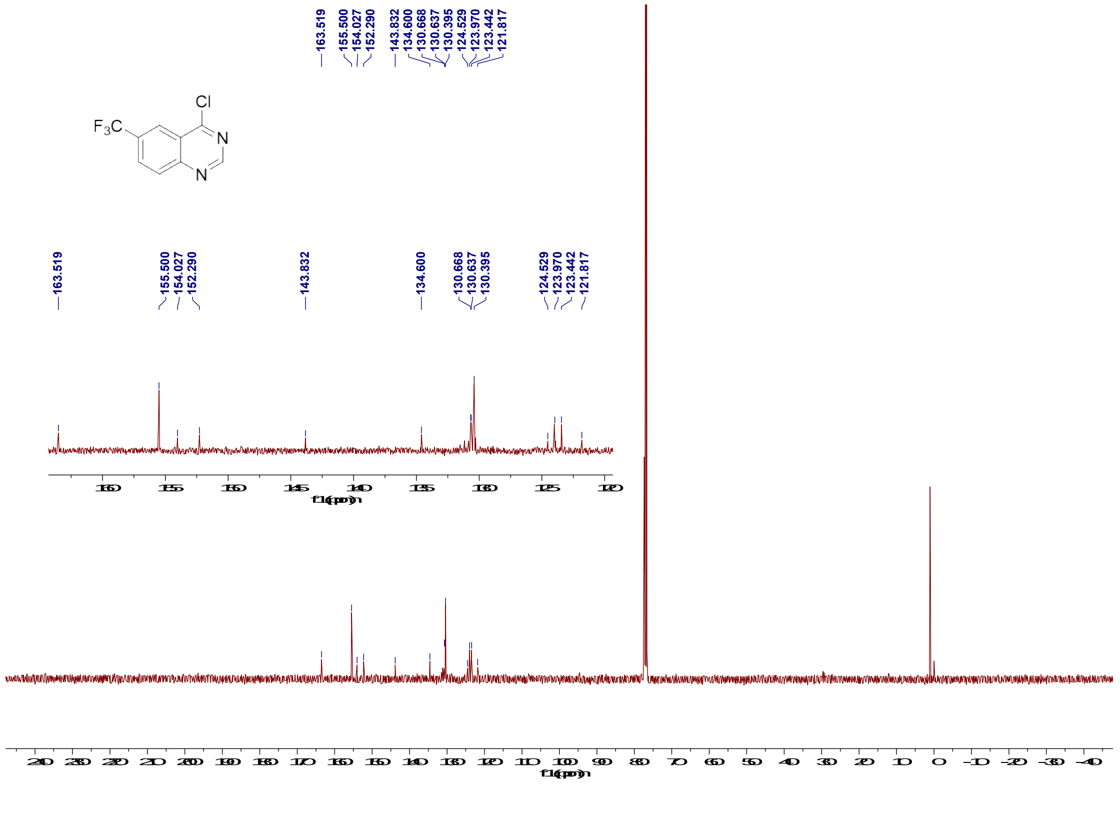

^{1}H NMR spectrum of 7-chloro-2-(trifluoromethyl)thieno[3,2-b]pyridine (4p) (400 MHz, $CDCl_3$)

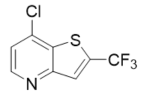

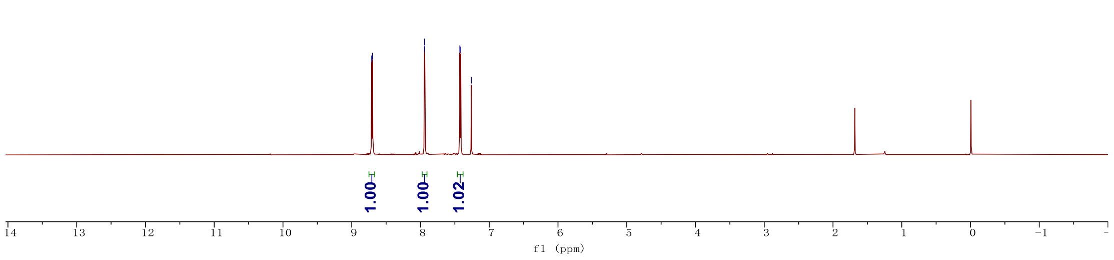

^{19}F NMR spectrum of 7-chloro-2-(trifluoromethyl)thieno[3,2-b]pyridine (4p)

(376 MHz, $CDCl_3$)

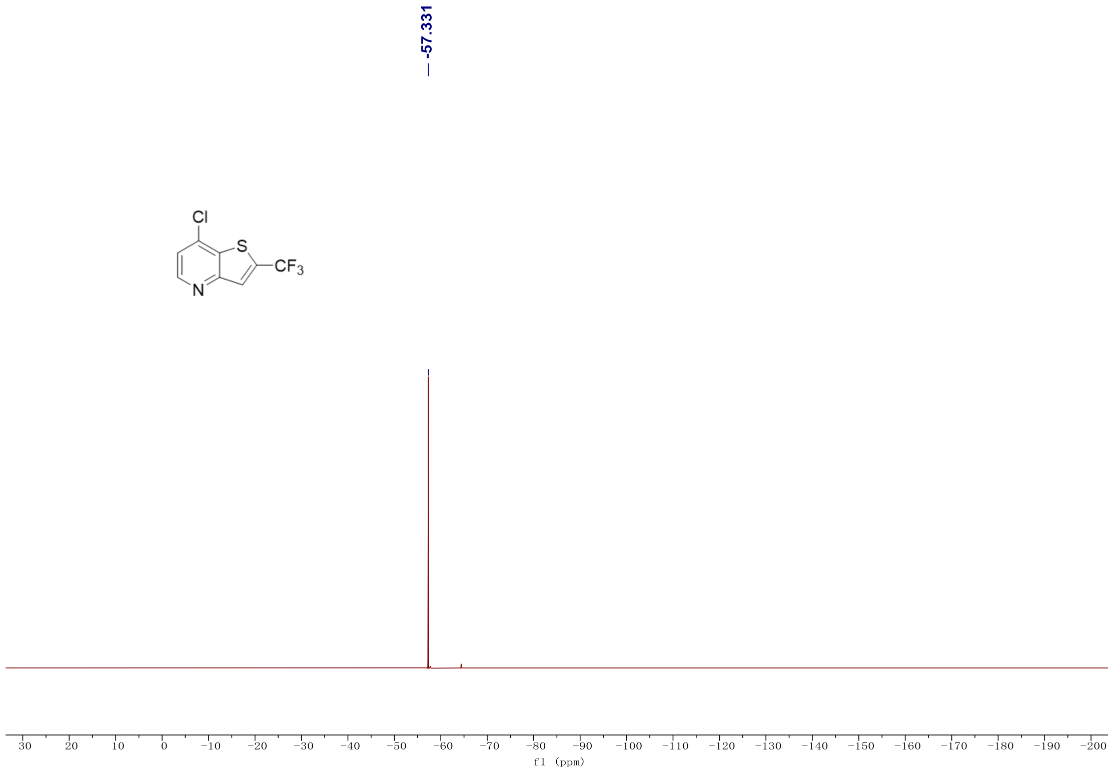

^{13}C NMR spectrum of 7-chloro-2-(trifluoromethyl)thieno[3,2-b]pyridine (4p)

(101 MHz, $CDCl_3$)

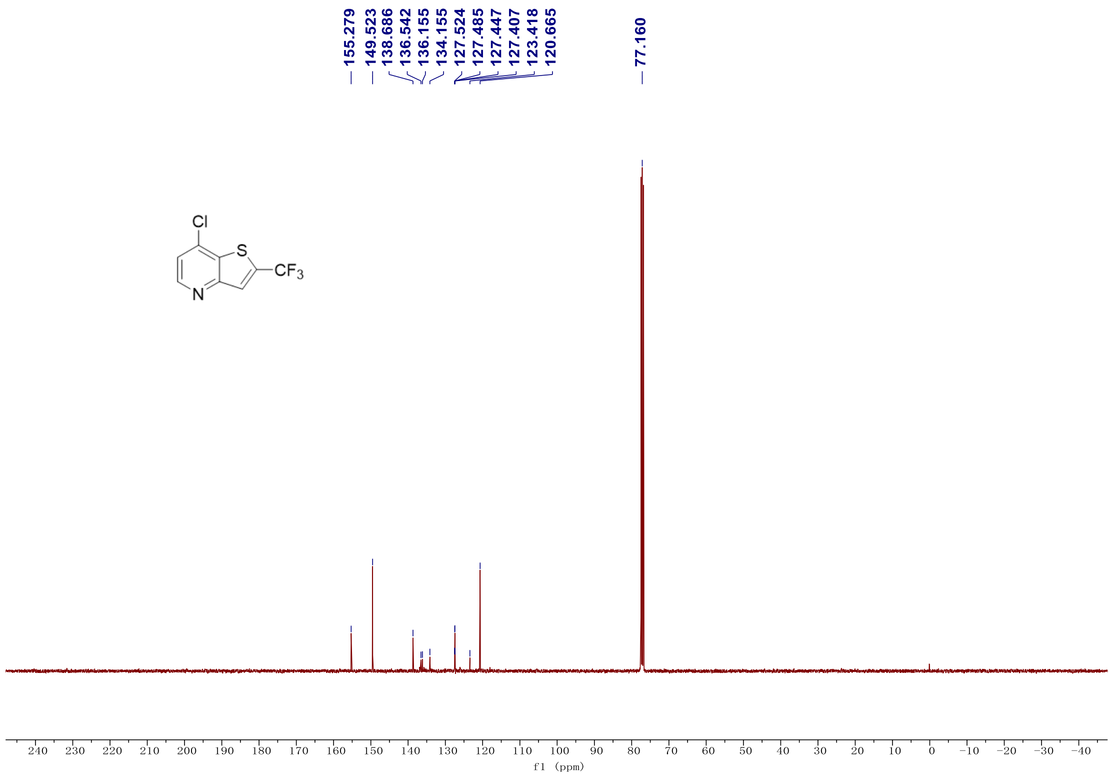

^{1}H NMR spectrum of 1-methoxy-3-(2,2,2-trifluoroethyl)benzene (4t) (400 MHz, $CDCl_3$)

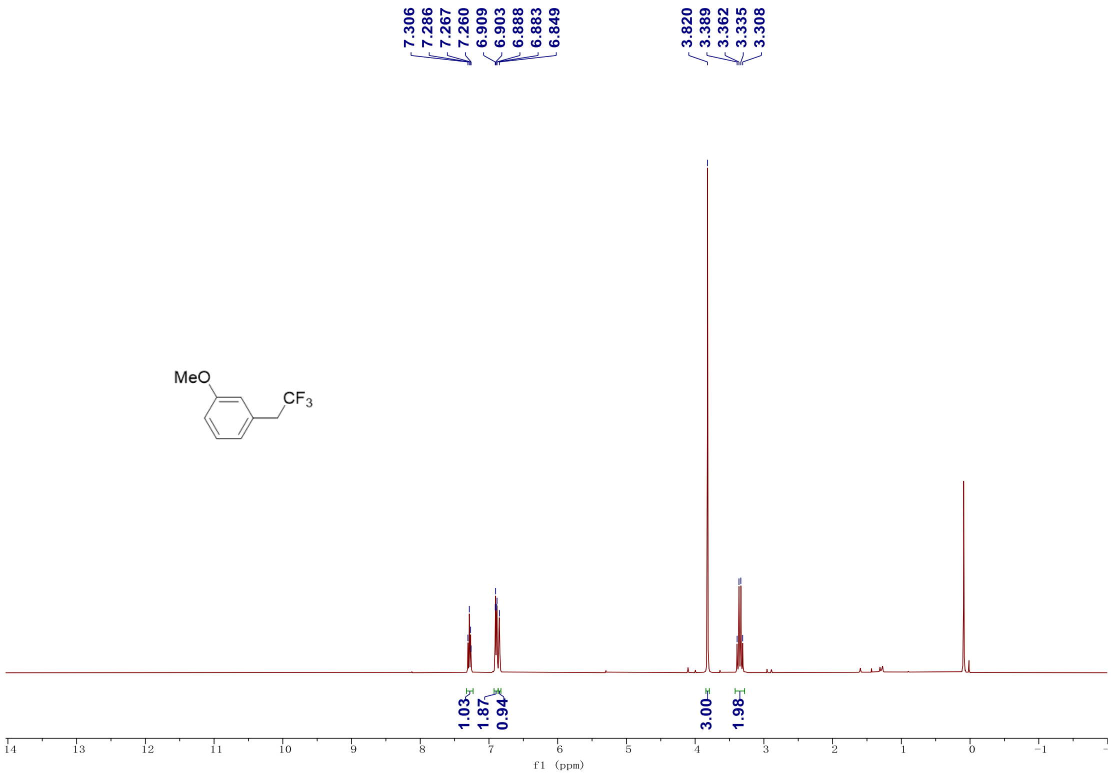

^{19}F NMR spectrum of 1-methoxy-3-(2,2,2-trifluoroethyl)benzene (4t) (376 MHz, $CDCl_3$)

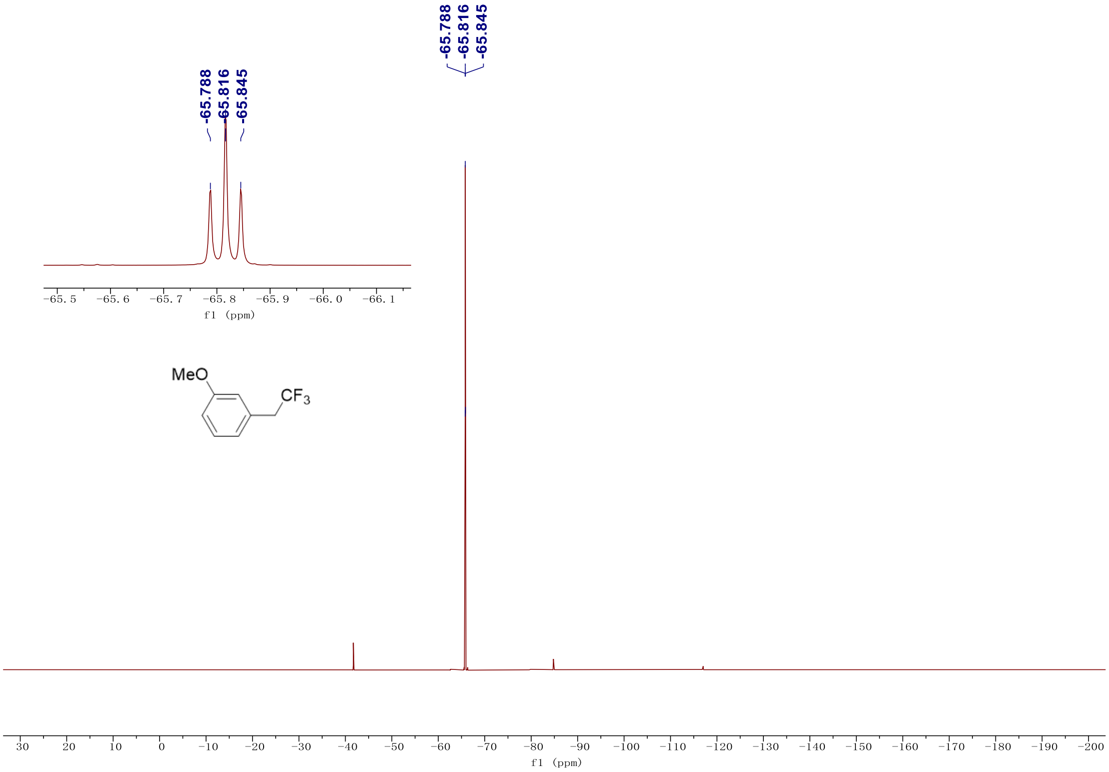

^{13}C NMR spectrum of 1-methoxy-3-(2,2,2-trifluoroethyl)benzene (4t) (101 MHz, CDCl₃)

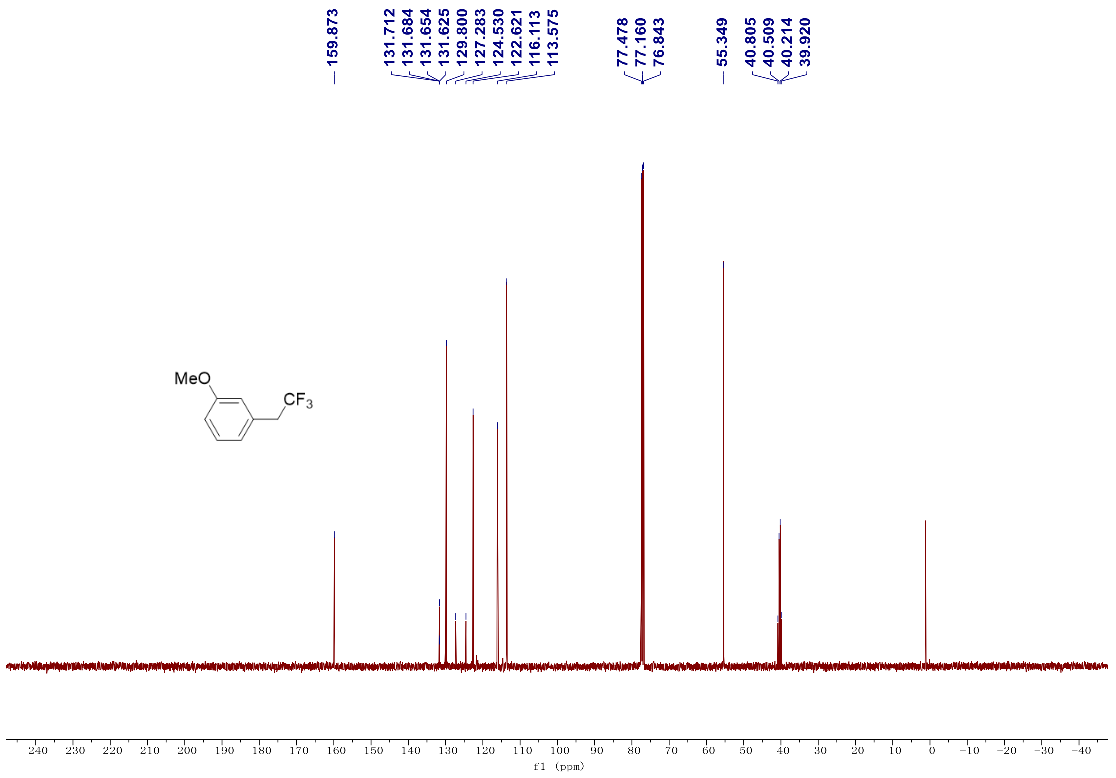

^{1}H NMR spectrum of 1,3-dimethoxy-5-(2,2,2-trifluoroethyl)benzene (4u) (400 MHz, CDCl₃)

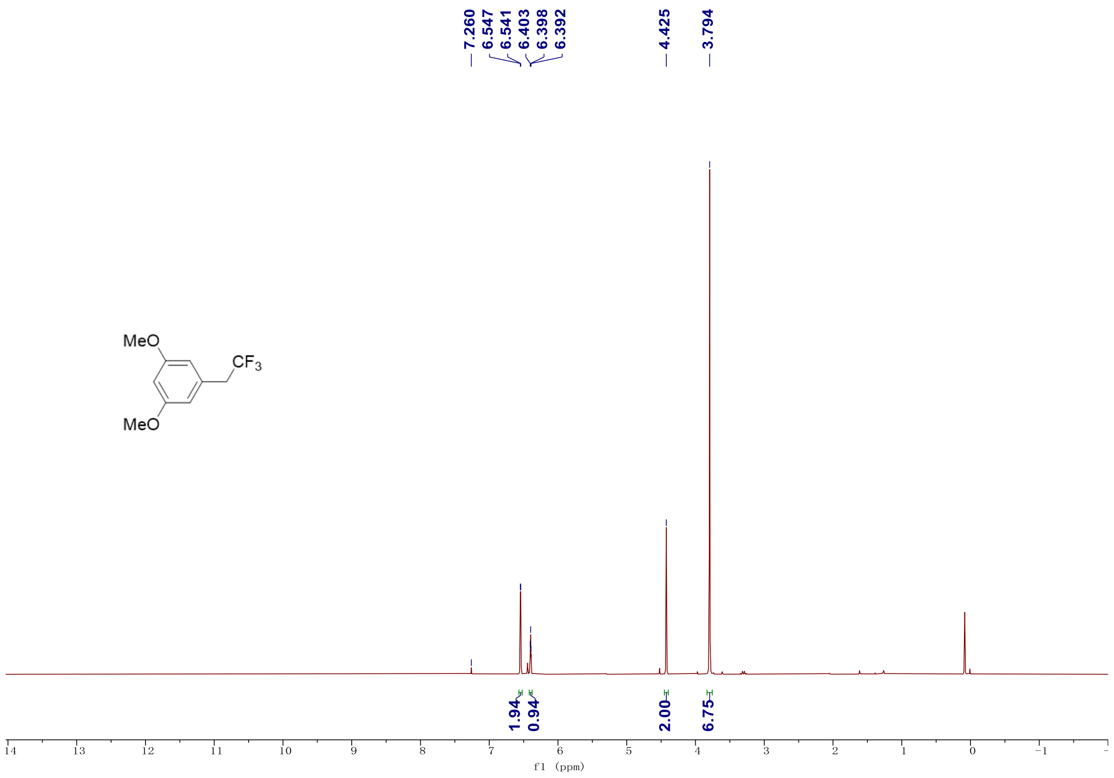
7.260
6.547
6.541
6.403
6.398
6.392
4.425
3.794
MeO
CF3
MeO
1.94
0.94
2.00
6.75
f1 (ppm)

^{19}F NMR spectrum of 1,3-dimethoxy-5-(2,2,2-trifluoroethyl)benzene (4u)

(376 MHz, $CDCl_3$)

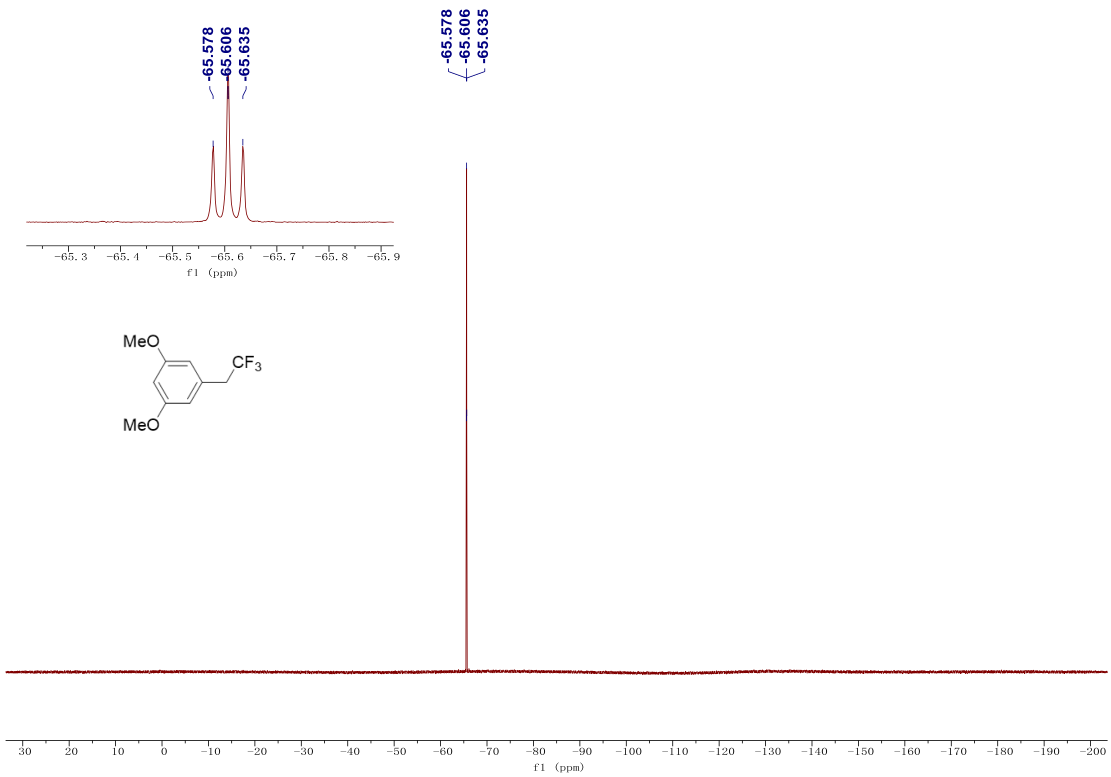

^{13}C NMR spectrum of 1,3-dimethoxy-5-(2,2,2-trifluoroethyl)benzene (4u)

(101 MHz, $CDCl_3$)

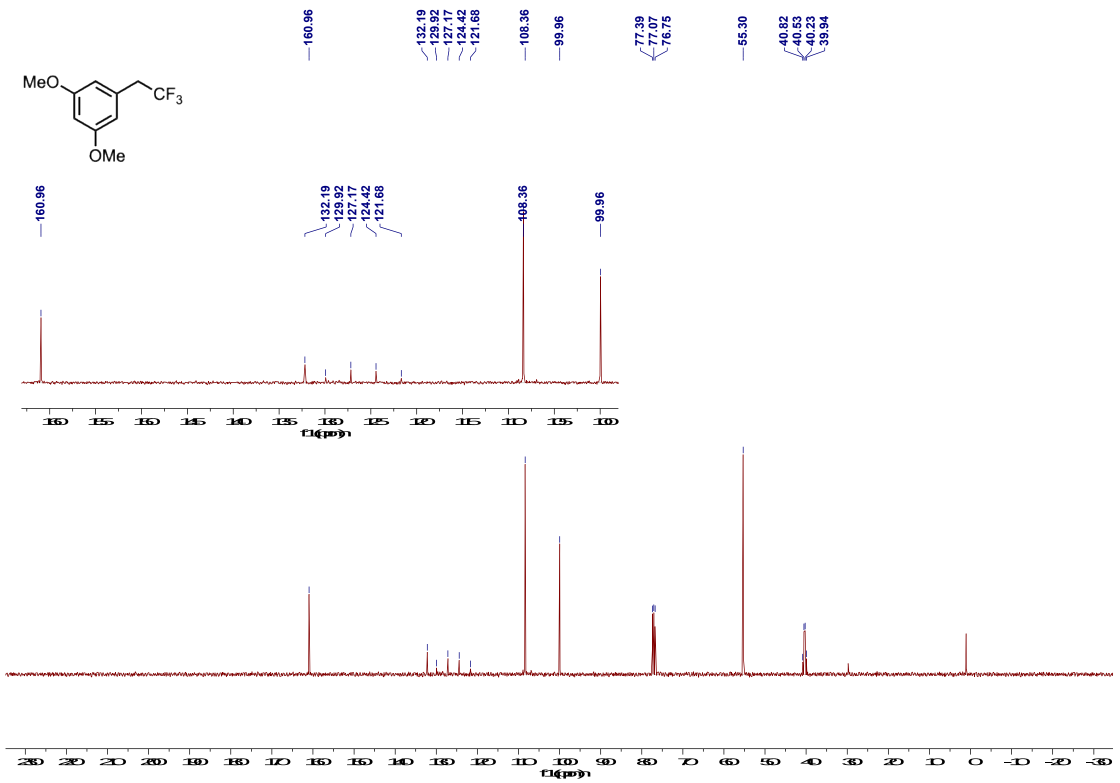

^{1}H NMR spectrum of 3-(2,2,2-trifluoroethyl)benzonitrile (4v) (400 MHz, $CDCl_3$)

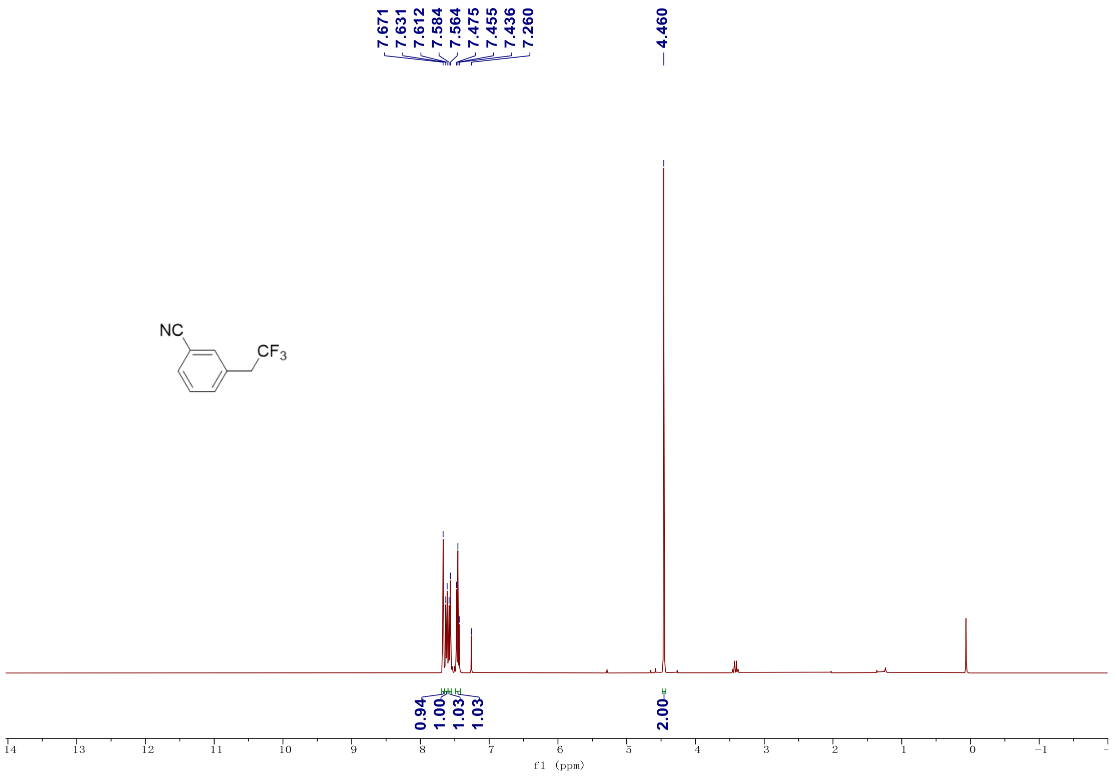

^{19}F NMR spectrum of 3-(2,2,2-trifluoroethyl)benzonitrile (4v) (376 MHz, $CDCl_3$)

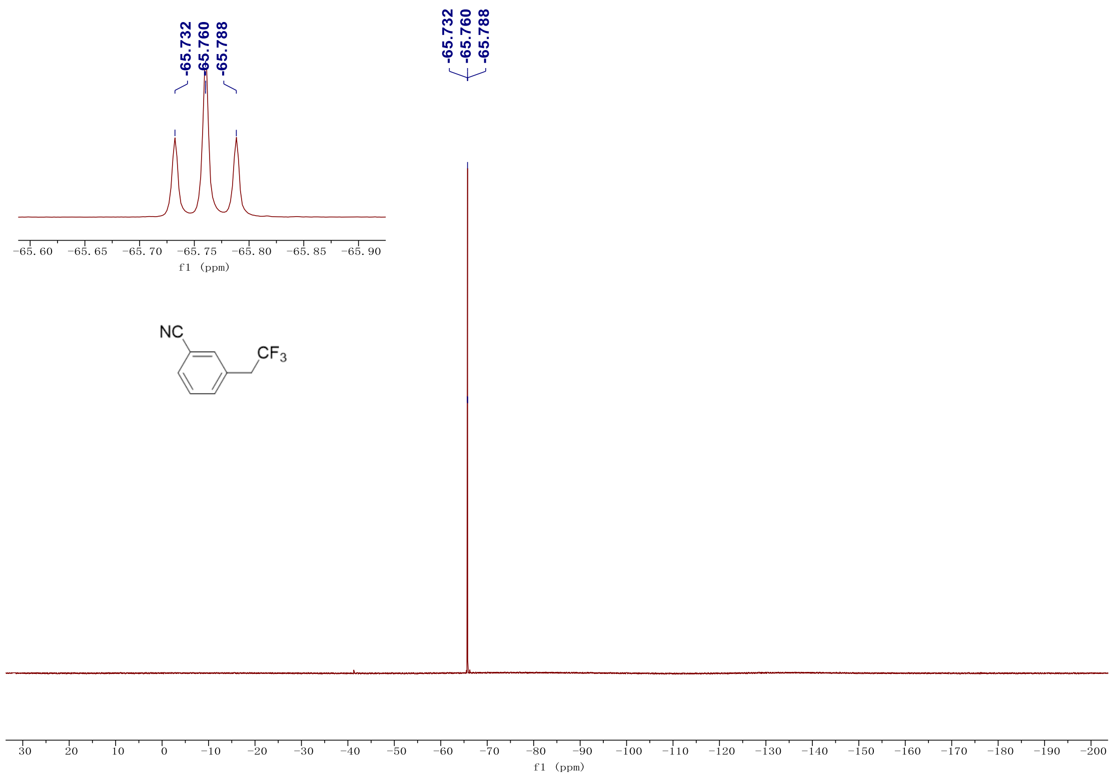

^{13}C NMR spectrum of 3-(2,2,2-trifluoroethyl)benzonitrile (4v) (101 MHz, $CDCl_3$)

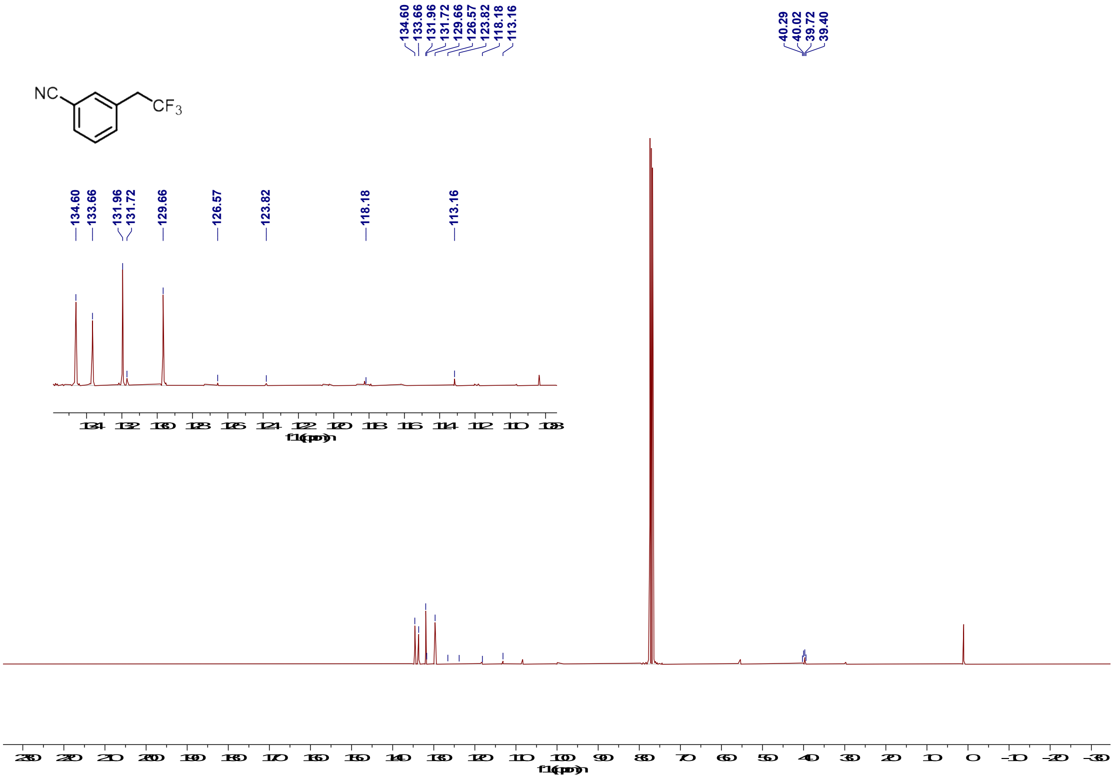

^{1}H NMR spectrum of 4-(2,2,2-trifluoroethyl)benzonitrile (4w) (400 MHz, $CDCl_3$)

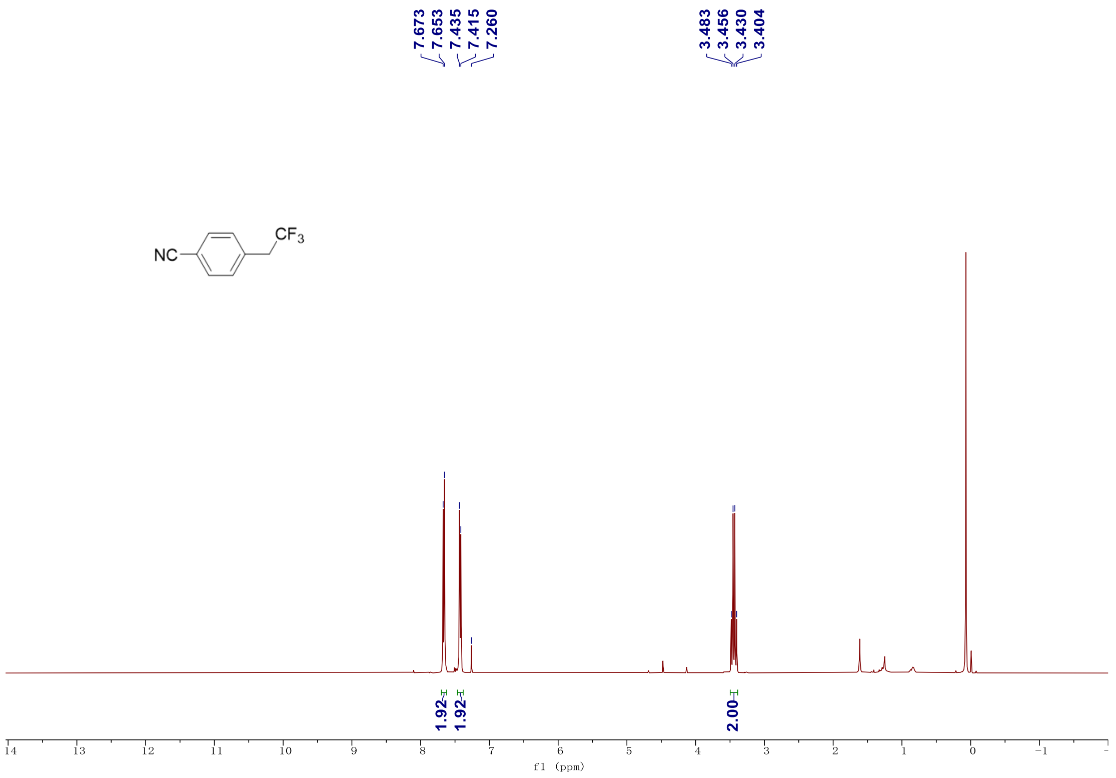
7.673
7.653
7.435
7.415
7.260
3.483
3.456
3.430
3.404
NC
CF3
1.92
1.92
2.00
14
13
12
11
10
9
8
7
6
5
4
3
2
1
0
-1
f1 (ppm)

^{19}F NMR spectrum of 4-(2,2,2-trifluoroethyl)benzonitrile (4w) (376 MHz, CDCl3)

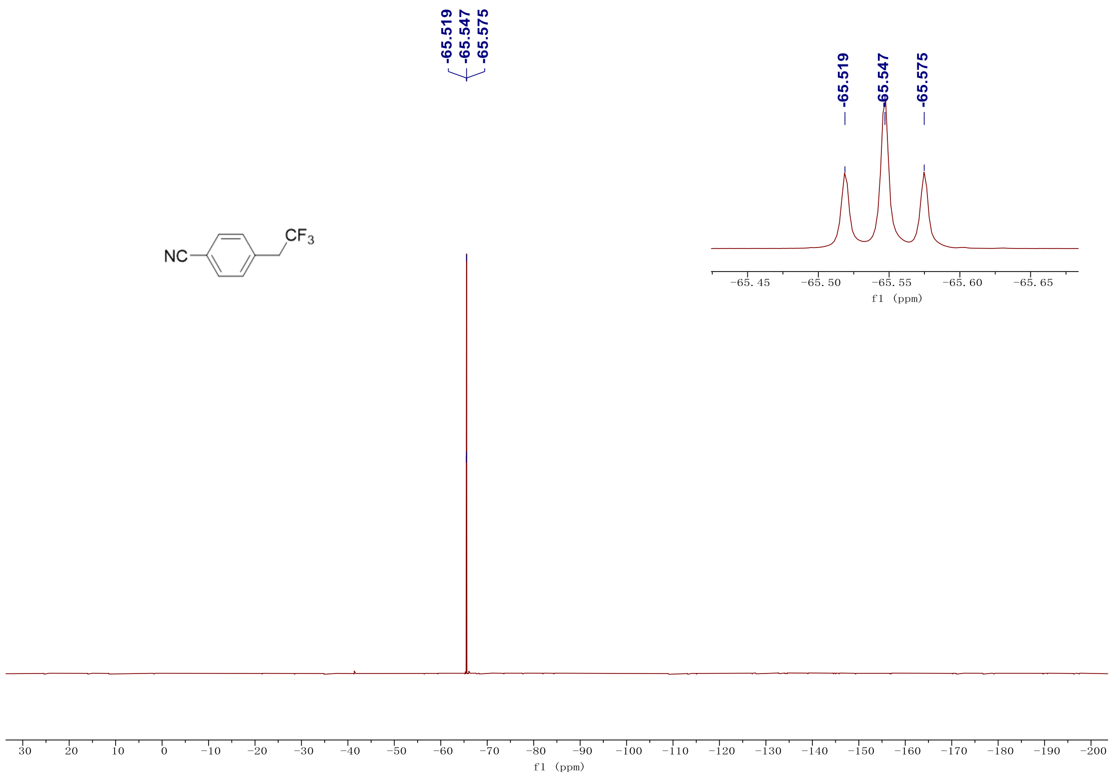

^{13}C NMR spectrum of 4-(2,2,2-trifluoroethyl)benzonitrile (4w) (101 MHz, CDCl3)

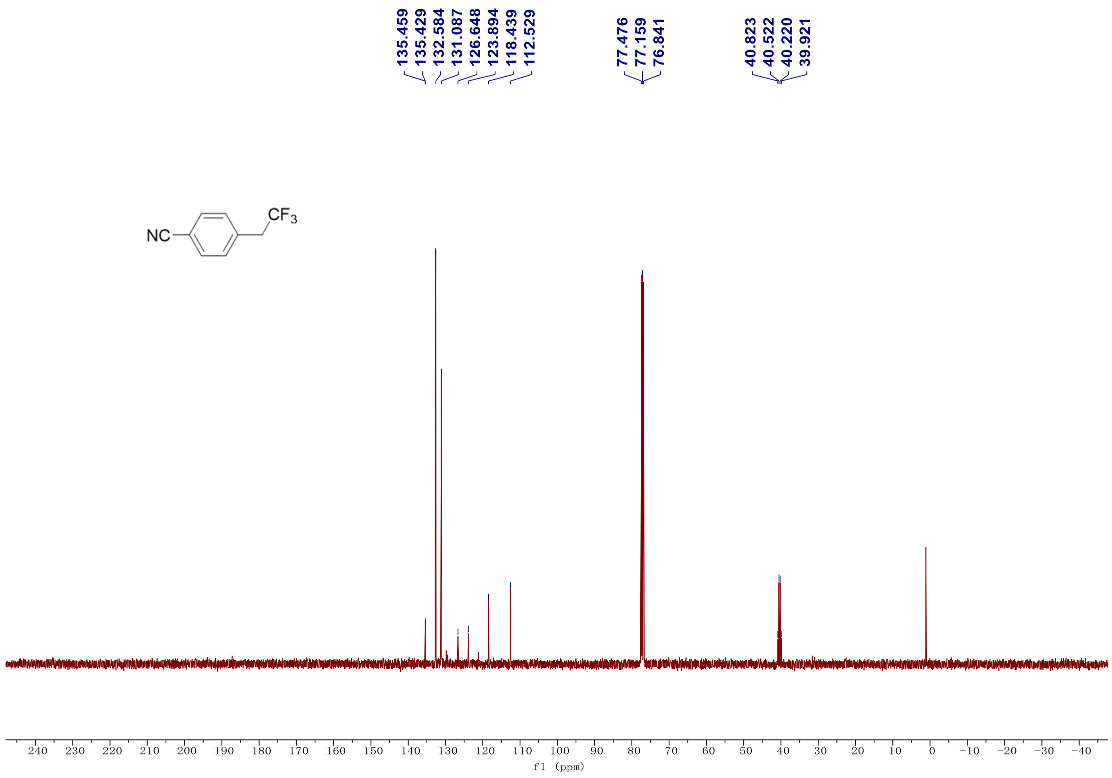

^{1}H NMR spectrum of 1-nitro-4-(2,2,2-trifluoroethyl)benzene (4x) (400 MHz, $CDCl_3$)

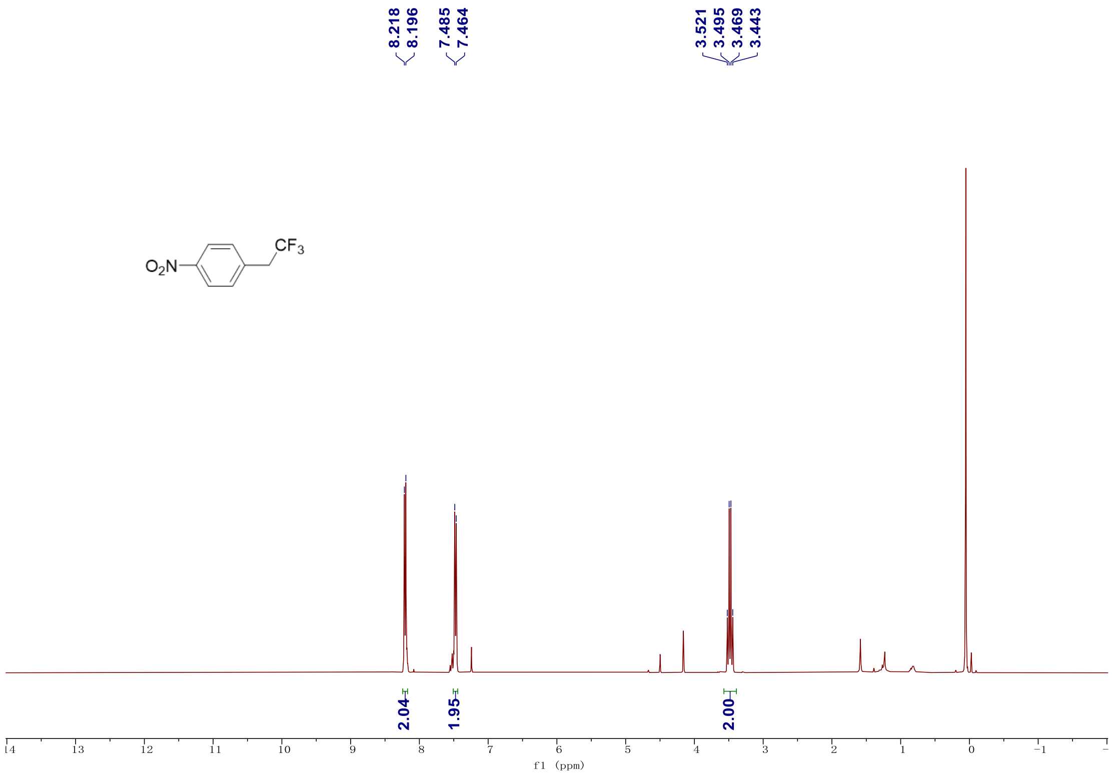

^{19}F NMR spectrum of 1-nitro-4-(2,2,2-trifluoroethyl)benzene (4x) (376 MHz, $CDCl_3$)

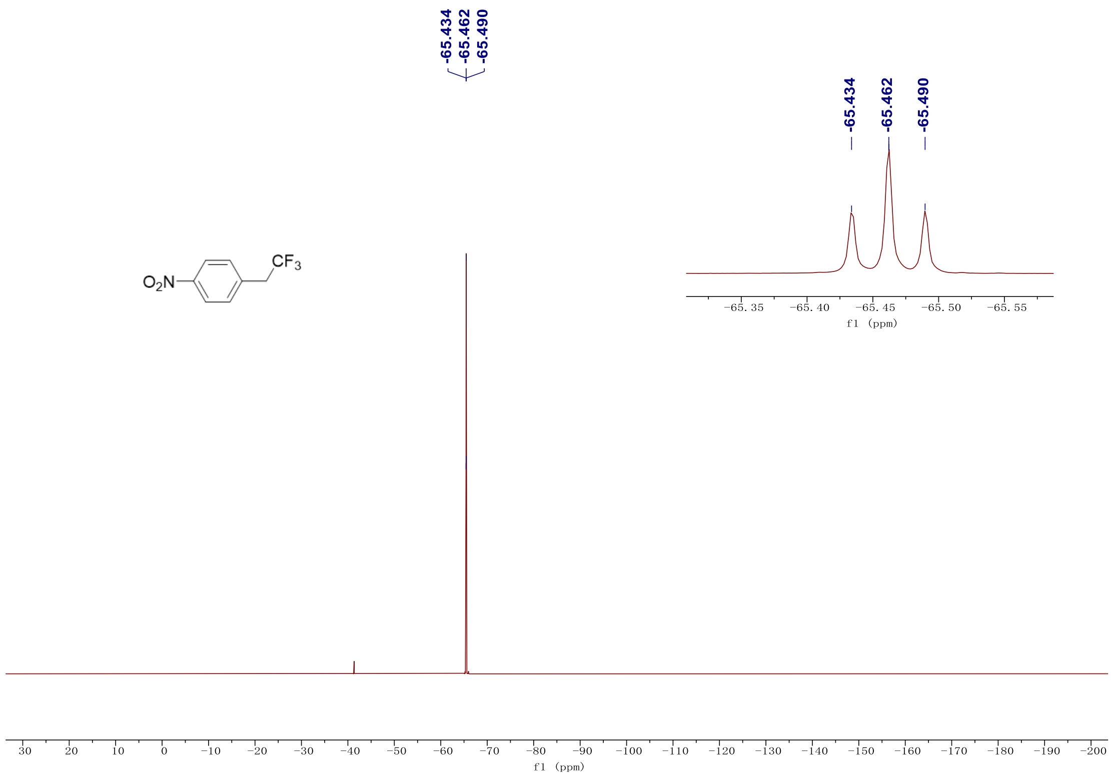

^{13}C NMR spectrum of 1-nitro-4-(2,2,2-trifluoroethyl)benzene (4x) (101 MHz, $CDCl_3$)

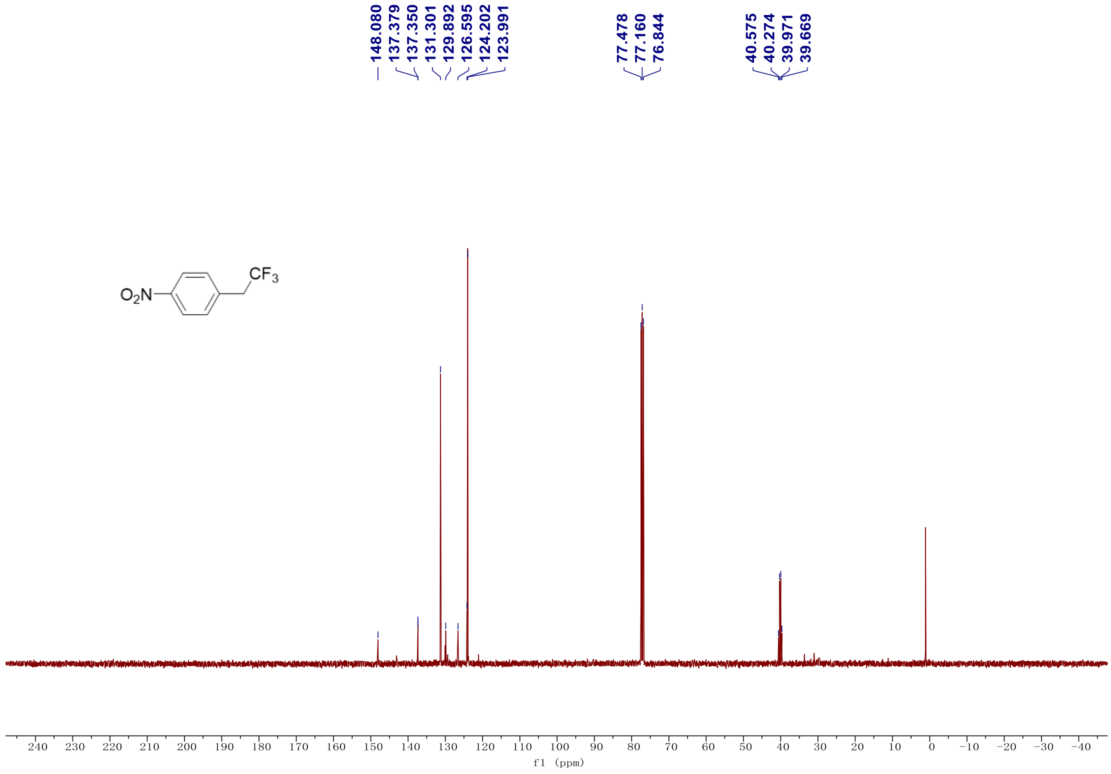
148.080
137.379
137.350
131.301
129.892
126.595
124.202
123.991
77.478
77.160
76.844
40.575
40.274
39.971
39.669
O2N
CF3
240 230 220 210 200 190 180 170 160 150 140 130 120 110 100 90 80 70 60 50 40 30 20 10 0 −10 −20 −30 −40
f1 (ppm)
